# Trans-histone crosstalk establishes distinct H3K79 methylation zones with differential transcriptional functions

**DOI:** 10.1093/nar/gkag291

**Published:** 2026-04-02

**Authors:** Na Hyun Park, Hwa-Ryeon Kim, Hye Young Kim, Soo Young Lee, Yong-Joon Cho, Jae-Seok Roe, TaeSoo Kim

**Affiliations:** Department of Life Sciences and Multitasking Macrophage Research Center, Ewha Womans University, Seoul 03760, Republic of Korea; Department of Biochemistry, College of Life Science and Biotechnology, Yonsei University, Seoul 03722, Republic of Korea; Department of Biomedical Sciences, Seoul National University College of Medicine, Seoul, Republic of Korea; Department of Life Sciences and Multitasking Macrophage Research Center, Ewha Womans University, Seoul 03760, Republic of Korea; Department of Molecular Bioscience, College of Biomedical Science, Kangwon National University, Chuncheon 24341, Republic of Korea; Multidimensional Genomics Research Center, Kangwon National University, Chuncheon 24341, Republic of Korea; Department of Biochemistry, College of Life Science and Biotechnology, Yonsei University, Seoul 03722, Republic of Korea; Department of Life Sciences and Multitasking Macrophage Research Center, Ewha Womans University, Seoul 03760, Republic of Korea

## Abstract

H3K79 methylation by Dot1 (disruptor of telomeric silencing-1) plays critical roles in multiple cellular processes potentially by modulating chromatin structure and gene expression. However, the genome-wide distribution patterns of H3K79me1, H3K79me2, and H3K79me3 and the mechanisms specifying these patterns remain unclear. Here, we mapped H3K79 methylation patterns across the yeast genome using ChIP-seq and identified three distinct gene groups, termed state-specific methylation zones, each predominantly marked by one methylation state. These zones remain largely stable during transcriptional reprogramming. They may be established and/or maintained via H2B ubiquitination by the Rad6–Bre1 complex: loss of Rad6 leads to the complete loss of the H3K79me3 zone, converting it into an H3K79me1-enriched region while simultaneously diminishing the H3K79me1 zone. Loss of H4K16 acetylation also similarly disrupted the H3K79me1 zone, albeit weakly. Interestingly, Dot1 occupancy does not always correlate with the H3K79me3 level, as translation-related genes exhibit high Dot1 occupancy but are depleted of H3K79me3. Functionally, H3K79me3 and H3K79me1 appear to play differential roles in transcriptional regulation: Dot1-activated genes are enriched for H3K79me3, whereas a majority of Dot1-repressed genes are associated with H3K79me1. We therefore propose that H3K79 methylation states define specific chromatin zones that contribute to differential transcriptional outputs and whose establishment and maintenance depend on trans-histone crosstalk.

## Graphical Abstract

**Graphical Abstract:**
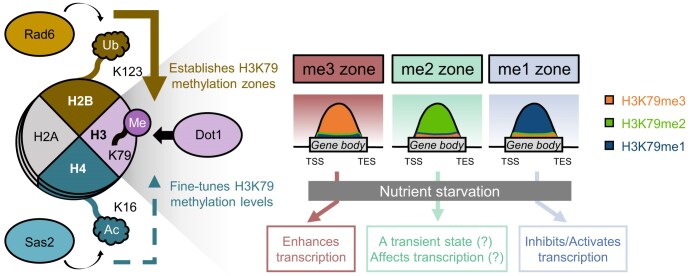

## Introduction

Histones undergo a wide range of post-translational modifications, including acetylation, methylation, phosphorylation, and ubiquitination, that play pivotal roles in the regulation of chromatin architecture and RNA polymerase II (RNA Pol II)-mediated transcription [1, 2]. Histone acetylation generally promotes transcription by disrupting histone–DNA interactions and loosening chromatin structure, whereas histone methylation provides binding platforms for downstream effectors that either activate or repress transcription. With regard to histone methylation, it occurs on specific lysine or arginine residues, and the functional outcome is highly residue specific. Moreover, unlike acetylation, lysine residues can be mono-, di-, or tri-methylated (me1, me2, and me3), each state often displaying distinct genomic distributions and biological functions [1, 3].

Histone H3 at K79 (H3K79) can be mono- (H3K79me1), di- (H3K79me2), or tri-methylated (H3K79me3). These methylation events are catalyzed by the H3K79 methyltransferase Dot1 (disruptor of telomeric silencing-1). Dot1 was first identified in yeast [4] and bears a catalytic domain that is structurally related to class-I methyltransferases [5, 6]. Although Dot1-mediated H3K79 methylation is evolutionarily conserved from yeast to humans [7–11], the H3K79 methylation landscape differs markedly between species. In yeast, ~90% of H3K79 is methylated, and H3K79me3 is the predominant form [8]. By contrast, in human cells, ~80% of H3K79 remains unmethylated, and H3K79me1 is the most abundant form (∼17%), followed by H3K79me2 (∼3%); H3K79me3 is very rare (<0.1%) [12]. The distribution patterns of methylated H3K79 also differ between species. In yeast, H3K79me3 is enriched over gene bodies, largely independently of steady-state transcription frequency [13]. By contrast, in humans, all three methylation states peak near the 5′ ends of actively transcribed genes, although variation between studies has been reported [12]. Why yeast and humans differ in the distribution is unclear at present but it may at least partly reflect mechanistic differences between Dot1 in yeast and its mammalian homolog DOT1L (Dot1-like): while it is still unclear how yeast Dot1 can be targeted to specific loci, DOT1L is known to interact with the phosphorylated C-terminal domain of elongating RNA Pol II through a domain that is only found in higher eukaryotes [14].

A previous study suggested that H3K79me3 and H3K79me2 in yeast exhibit distinct genome-wide distributions, although the pattern of H3K79me1 remains unclear [15]. The reason for this is not well understood. One possibility is that it reflects trans-histone crosstalk, which in turn influences Dot1 recruitment and/or activity. This is suggested by two observations: (i) Rad6-mediated H2B ubiquitination (H2Bubi) (H2BK123 in yeast, H2BK120 in mammals) is required for the deposition of H3K79me2 and H3K79me3 but has a lesser effect on H3K79me1 [16–21]; and (ii) H3K79 methylation by Dot1 can be promoted by acetylation of histone H4 at K16 (H4K16) [22]. Notably, recent structural studies suggest that H2Bubi and H4K16 acetylation (H4K16ac) collaboratively promote Dot1 activation via allosteric regulation [23, 24]. H3K79 methylation patterns may also be shaped by crosstalk with other histone modifications that antagonize H3K79 methylation, including phosphorylation of H3 at threonine 11 by SESAME (Serine-responsive SAM-containing Metabolic Enzyme) and histone deacetylation by Rpd3/HDAC1 [25, 26]. Thus, it is likely that multiple histone post-translational modifications coordinate to shape the deposition of the three H3K79 methylation states throughout the genome [24]. However, the details of this mechanism remain to be elucidated.

H3K79 methylation is an important epigenetic modification that has been linked to many processes, including telomeric silencing [8], cell cycle checkpoint control [27], the DNA damage response [28, 29], and developmental processes [30]. However, whether these links reflect the modulation of RNA Pol II transcription by H3K79 methylation [12, 31] is not clear, in part because specific effector proteins that directly recognize H3K79 methylation have not yet been identified [32, 33]. Notably, transcriptome analyses indicate that global mRNA levels are largely unaffected by the loss of Dot1 or H3K79 methylation, but these analyses focus on cells in steady-state conditions [22, 34]. Given that chromatin regulators often influence the kinetics of transcriptional responses to environmental cues [2, 34–37], Dot1-dependent H3K79 methylation may play a more critical role in dynamic transcriptional responses rather than in maintaining basal gene expression. Thus, to understand the relationship between H3K79 methylation and RNA Pol II transcription, it is important to elucidate the function of Dot1 and the H3K79 methylation landscape during transcriptional reprogramming induced by environmental changes.

In this study, we generated genome-wide maps of H3K79me1, H3K79me2, and H3K79me3 in yeast and revealed that each methylation state predominantly marks distinct, non-overlapping subsets of genes. These subsets were termed “state-specific H3K79 methylation zones”. We also found that these zones remained remarkably stable during nutrient starvation-induced transcriptional reprogramming. Moreover, we observed that the establishment of these zones depended on Rad6-mediated H2Bubi, since deletion of *RAD6* results in loss of the H3K79me3 zone (me3 zone), leading to its conversion into an H3K79me1-enriched region. Interestingly, Rad6-mediated H2Bubi was also required for the establishment of the H3K79me1 zone (me1 zone). However, H2Bubi is unlikely to promote chromatin binding of Dot1. H3K79 methylation patterns were also fine-tuned by H4K16 acetylation, since loss of H4K16ac slightly changed the distribution of H3K79 methylation. These findings suggest that multiple forms of trans-histone crosstalk are important for establishing the distribution of the three H3K79 methylation states. Interestingly, at a subset of genes associated with translation and high expression levels, lower methylation states, but not H3K79me3, were enriched despite exhibiting the highest levels of Dot1 binding, H2Bubi, and RNA Pol II occupancy. In addition, H3K79me1 and H3K79me3 likely regulate gene expression in differential, context-dependent ways. Whereas Dot1 is required for transcription of genes within the H3K79me3 zone, a majority of Dot1-repressed genes are associated with H3K79me1. Thus, each H3K79 methylation state defines a specific and functionally distinct chromatin zone, and the establishment of these zones depends on trans-histone crosstalk.

## Materials and methods

### Yeast strains

The yeast strains used in this study are listed in Supplementary Table S1. The gene deletion mutants were constructed by homologous recombination of PCR fragments according to standard protocols [38]. Dot1-FLAG strains were generated by inserting the 3× FLAG tag into the C-terminus of *DOT1*. The 3× FLAG tag sequence was amplified with pFA6a-6×GLY-3×FLAG-kanMX6 (Addgene plasmid # 20 754) by using primers. The sequences of oligonucleotides used in this study are listed in Supplementary Table S2.

### Yeast culture conditions

For most experiments, yeast cells were grown in YPD [yeast extract peptone dextrose: 1% yeast extract, 2% peptone, 2% dextrose (glucose)] at 30°C until the OD_600 nm_ was 0.5–0.6. For nutrient starvation, cells were grown in YPD at 30°C until the OD_600 nm_ was 0.5–0.6, after which half were transferred to 0.15× YP (0.15% yeast extract and 0.3% peptone) for 4 h at 30°C.

### Antibodies

Antibodies were obtained from the following sources: anti-H3 (1:10000; ab1791), anti-H3K79me3 (1:2500; ab2621), and anti-H3K79me2 (1:5000; ab3594) from Abcam; anti-H3K79me1 (1:1000; 39145) from Active Motif; and anti-FLAG M2 (1:3000; F1804-1MG) from Sigma-Aldrich. Synthetic peptides (Anaspec) corresponding to unmodified H3 (residues 69–83), H3K79me1 (69–89), H3K79me2 (69–89), and H3K79me3 (69–89) were used for antibody validation.

### Western blot analysis

Cells were grown in YPD at 30°C to mid-log phase and lysed in buffer (50 mM Tris, pH 7.5, 150 mM NaCl, 0.1% NP-40) with protease inhibitors [2 μM pepstatin A, 0.6 μM aprotinin, 2 μM leupeptin, 1 mM phenylmethylsulfonyl fluoride (PMSF)] and glass beads (Sigma). Protein concentration was measured by Bradford assay. For sodium dodecylsulfate (SDS)–polyacrylamide gel electrophoresis (PAGE) and western blot analyses [39], 20 μg of whole-cell extracts were used. Proteins were separated by SDS–PAGE and transferred to a nitrocellulose blotting membrane (Cytiva). After blocking, membranes were incubated with the primary antibody and then washed with phosphate-buffered saline–Tween (PBST). After incubation with horseradish peroxidase (HRP)-conjugated secondary antibody, the membranes were washed three times with PBST. The blots were developed using Super Signal West Pico Chemiluminescent Substrate (Thermo Fisher Scientific) and imaged on film (AGFA).

### Chromatin immunoprecipitation and ChIP-sequencing

Chromatin immunoprecipitation (ChIP) was conducted as previously described [40, 41] with oligonucleotides listed in Supplementary Table S2. Cells were subjected to 1% formaldehyde cross-linking, sonication, and immunoprecipitation with the relevant antibodies. Anti-H3, anti-H3K79me3, anti-H3K79me2, and anti-H3K79me1 were induced to bind to Protein A agarose beads at 4°C overnight in FA lysis buffer containing 275 mM NaCl. For anti-FLAG M2 antibody, Protein G Dynabeads (Invitrogen) were used for antibody binding. Precipitates were washed with the same buffer, then once with FA lysis buffer containing 500 mM NaCl, once with 10 mM Tris–HCl (pH 8.0), 0.25 M LiCl, 1 mM EDTA, 0.5% NP-40, 0.5% Na deoxycholate, and once with TE [10 mM Tris–HCl (pH 8.0), 1 mM EDTA]. Precipitated DNAs were analyzed by real-time quantitative PCR (qPCR) using CFX96 cycler (Bio-Rad) and THUNDERBIRD SYBR qPCR Mix (TOYOBO). ChIP-sequencing (ChIP-seq) libraries for genome-wide sequencing were prepared using the Accel-NGS 2S Plus DNA Library Kit (Swift BioSciences, 21096) according to the manufacturer’s instructions. The prepared libraries were sequenced on a HiSeq2500 platform (Illumina) using the paired-end method (101 bp reads).

### ChIP-seq data analysis

ChIP-seq analyses were performed as previously described [42]. Briefly, paired-end 101 bp sequencing reads were mapped to the reference *Saccharomyces cerevisiae* (sacCer3) genome assemblies using Bowtie2 with default settings, and duplicated mapped reads were removed using SAMtools. The HOMER suite was used to identify peaks from each H3K79me3, me2, and me1 ChIP-seq dataset and combined to identify total union H3K79 methylation peaks using default settings (-d given, *n* = 42788). The ChIP-seq tag counts of each H3K79 methylation state were then recalculated at a union of 42788 intervals and normalized to a read depth of 10 million uniquely mapped reads. Gene annotation of ChIP-seq reads was performed using annotatePeaks.pl in the HOMER suite with default settings. For visualization with the Integrative Genomics Viewer (IGV) [43–45], the makeBigWig tool (in the HOMER suite) was used to generate bigWig files. After calculating the mean RPM (reads per million) values of ChIP-seq signals for H3K79me3, H3K79me2, H3K79me1, and H3 of each gene, heatmaps were drawn with the guidance of EaSeq. To draw the heatmaps, the genes were sorted according to the mean RPM value of H3K79me3 of the wild-type (WT) strain, mapped from 1 kb upstream of the transcription start site (TSS) to 1 kb downstream of the transcription end site (TES). The R packages (ver. 3.4.1) (http://www.r-project.org) [46] were used to draw a heatmap and scatterplot of gene body enrichment of H3K79me3, me2, and me1 (1 kb window resolution) for genes assigned to each zone. Average plots were generated using deepTools (computeMatrix and plotProfile, version 3.5.4) implemented on the Galaxy platform (https://usegalaxy.eu) [47]. Normalized bigWig files were used as input together with the list of target genes. The signal matrix was computed over the gene body regions with ± 1 kb flanking sequences, and average plots were visualized. Gene Ontology (GO) enrichment analysis was performed using YeastEnrichr [48].

### RNA sequencing

WT and *dot1Δ* strains were grown in YPD to an OD_600_ of 0.5–0.6, after which half of each culture was shifted to 0.15× YP for 4 h. Total RNA was extracted from 50 ml of cells using the hot phenol method, followed by DNase I treatment (Thermo Scientific Fisher). RNA sequencing libraries were prepared using the TruSeq Stranded Total RNA Library Prep Kit (Illumina) after rRNA was depleted using the Ribo-Zero yeast kit (Epicenter), as recommended by the manufacturers. Paired-end sequencing of the 101-mer read length was carried out on an Illumina NextSeq 500 system. The resulting reads were then aligned to the *S. cerevisiae* S288C (sacCer3) reference genome using the HISAT2 mapping tool. Structural RNA was masked, and differentially expressed genes were identified using Cuffdiff. Differentially expressed genes were analyzed using the Cufflinks tools. All the following analyses were performed on genes with an RPKM (reads per kilobase of transcript per million mapped reads) value >0 in either control or experimental samples. Data visualization, including volcano plots and heatmaps, was performed using R packages (v3.4.1) [46, 49]. BigWig files were generated using the bamCoverage tool from deepTools (version 3.5.4) via the Galaxy platform (https://usegalaxy.eu) [47].

## Results

### The three H3K79 methylation states define distinct chromatin zones

Post-translational modifications of histones frequently exhibit specific genomic distributions that contribute to distinct regulatory outcomes. This may also be true for the three methylation states (mono-, di-, and tri-) of single lysine residues in histones. Indeed, the three methylation states of H3K4 and H3K36 are enriched in the genome in non-uniform patterns [50–53]. However, it is unclear whether H3K79 methylation also demonstrates such state-specific regional organization.

**Figure 1.**
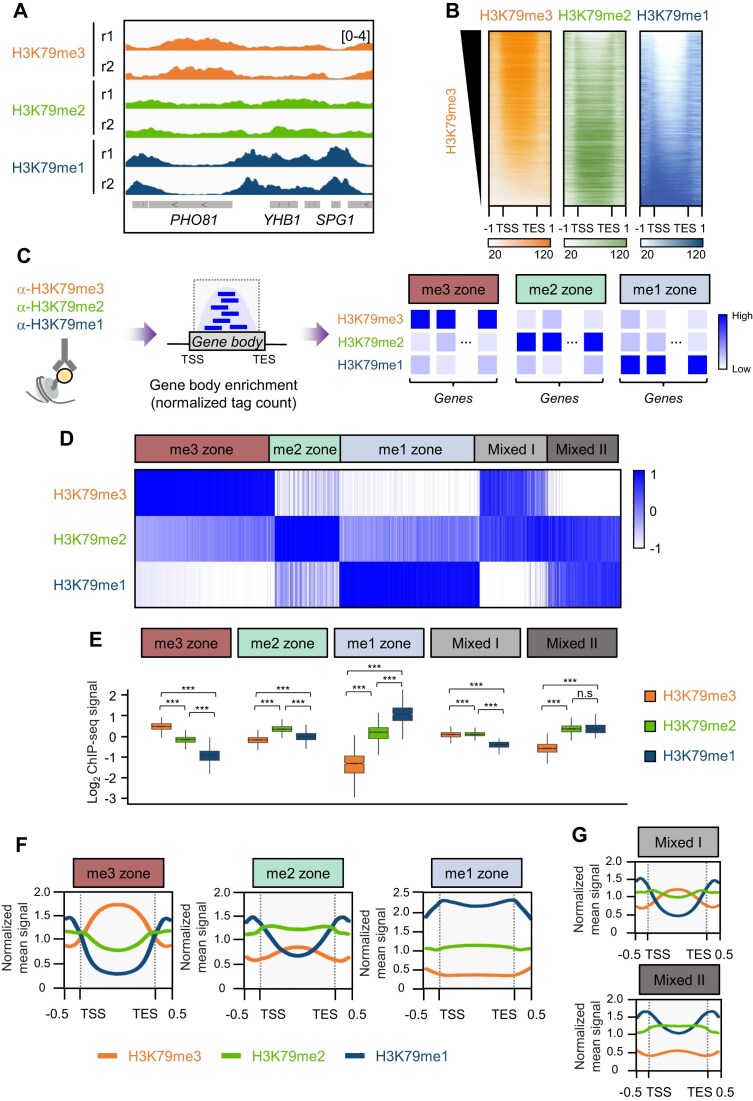
H3K79 methylation states define distinct chromatin zones. (**A**) Genome browser tracks for H3K79me3 (orange), H3K79me2 (green), and H3K79me1 (dark blue) from two independent biological replicates (r1 and r2), illustrating state-specific enrichment over different gene bodies. The H3K79 methylation levels were normalized to total histone H3 levels. (**B**) Heatmaps of ChIP-seq signals for all yeast genes (*n* = 5750), sorted in descending order of H3K79me3 signals. The averages of two biological replicates are shown. The *y*-axis lists the genes. The *x*-axis spans from the TSS to the TES and their 1 kb flanking regions. The color scale (RPM) ranges from 20 to 120. The color scheme is the same as that in (A). (**C**) Schematic depiction of zone classification. Genes were assigned to the me3, me2, or me1 zone if the read density of that state exceeded the read densities of both of the other two states by log_2_FC >0.25 and FDR *q* < 0.5. Genes not meeting these criteria are categorized as Mixed I (me2 ≈ me3 > me1) and Mixed II (me1 ≈ me2 > me3). (**D**) Heatmaps of the gene body enrichment of H3K79me3, me2, and me1 at the genes that had been classified to each zone. (**E**) Box plots comparing the log_2_ ChIP-seq signals of the three methylation states (H3K79me/H3) within the bodies of the genes in each zone/Mixed group. Statistical significance was assessed with a two-sided Wilcoxon rank-sum test (***P < 0.001, n.s: not significant). (**F** and **G**) Average profiles of normalized mean ChIP-seq signals for each methylation state in the three zones (F) and in the Mixed I and Mixed II groups (G). The shaded areas indicate the gene bodies from the TSS to the TES.

To address this question, we performed ChIP-seq for H3K79me3, H3K79me2, and H3K79me1 in WT *S. cerevisiae*, using state-specific antibodies that we independently validated for specificity and sensitivity (Supplementary Fig. S1A). Genome browser visualization showed that each methylation state localized preferentially at different gene bodies. For example, H3K79me3, H3K79me2, and H3K79me1 were particularly enriched at the gene bodies of *PHO81, YHB1*, and *SPG1*, respectively (Fig. 1A). The localization of H3K79me3 to coding regions is consistent with earlier observations [15, 22]. These findings indicate that all three methylation states of H3K79 can occupy gene bodies, but they do so at different loci.

To systematically assess the global distribution of these marks, we ranked all annotated yeast genes (*n* = 5750) by H3K79me3 occupancy and plotted the normalized read density for all three methylation states across a ± 1 kb window centered on the gene bodies (Fig. 1B). This comparative metagene analysis confirmed that H3K79me3, H3K79me2, and H3K79me1 were preferentially enriched at largely non-overlapping gene subsets, suggesting that H3K79 methylation states mark distinct chromatin zones.

To quantify these patterns, we calculated the gene body ChIP-seq tag counts of each H3K79 methylation state and systematically assigned each gene to the “me3”, “me2”, or “me1” zone if one state significantly exceeded both of the other states, as determined by pairwise comparisons using a log_2_ fold-change (FC) threshold of > 0.25 with multiple testing correction [false discovery rate (FDR) *q *< 0.5] (Fig. 1C). For example, a gene was classified as belonging to the me3 zone if H3K79me3 read density was greater than that of both H3K79me2 and H3K79me1, with log_2_FC (H3K79me3/H3K79me2) > 0.25 and log_2_FC (H3K79me3/H3K79me1) > 0.25, both at FDR *q* < 0.5. This classification revealed that there were 1649 genes in the me3 zone, 764 in the me2 zone, and 1617 in the me1 zone (Supplementary Table S3). The remaining genes, which did not meet these thresholds, were grouped into a “Mixed” category (Fig. 1D). Using alternative log_2_FC thresholds (0.1 or 0.5) only altered the number of genes assigned to each zone, without affecting the overall qualitative zonal patterns (Supplementary Fig. S1B).

Quantitative comparison of the zones confirmed the robustness of this classification: the dominant methylation state was strongly enriched in each zone, while the signals from the other states were significantly lower. Specifically, the median log_2_FC between the most enriched and second most enriched states was 0.778 for H3K79me3 versus H3K79me2 in the me3 zone, 0.443 for H3K79me2 versus H3K79me1 in the me2 zone, and 0.897 for H3K79me1 versus H3K79me2 in the me1 zone (Fig. 1E). Analysis of quantitative relationships among the H3K79 methylation states within each zone revealed a strong anti-correlation between H3K79me3 and H3K79me1 in the me3 or me1 zone. In contrast, in the me2 zone, H3K79me1 and H3K79me3 were present at comparable levels (Fig. 1E; Supplementary Fig. S1C). On analyzing the Mixed category, two distinct patterns emerged: the “Mixed I” genes displayed comparable H3K79me2 and H3K79me3 enrichment that both exceeded H3K79me1 levels, whereas the “Mixed II” genes exhibited similar levels of H3K79me2 and H3K79me1 that were both higher than H3K79me3 (Fig. 1D, E; Supplementary Fig. S1C). These observations suggest that the Mixed class may represent transitional or combinatorial states where no single methylation form predominates.

We next investigated whether the distribution of methylation across gene bodies differed between zones. Indeed, each zone displayed distinct spatial patterns. In the me1 zone, H3K79me1 displayed a strong broad peak at the coding regions, whereas H3K79me2 and H3K79me3 were present throughout at much lower levels. By contrast, in the me3 zone, H3K79me3 was tightly localized at the mid-coding regions, H3K79me1 was absent in the gene body but present at relatively high levels in the 5′- and 3′-flanking regions, and H3K79me2 was moderately distributed across the gene body. The me2 zone demonstrated a somewhat intermediate pattern, with a broad peak of H3K79me2 at the coding regions and overall profiles of H3K79me1 and H3K79me3 similar to those observed in the me3 and me1 zones, respectively (Fig. 1F). Mixed I resembled the me3 zone but with lower gene body H3K79me3 levels, while Mixed II resembled the me2 zone but with lower gene body H3K79me2 levels (Fig. 1G). Thus, each zone is defined not only by dominance of a single methylation state but also by characteristic distributional signatures of the non-dominant states. Collectively, these analyses show that H3K79 methylation states distinguish distinct chromatin zones, each characterized by a primary mark and unique secondary distributional features.

### H2Bubi and H4K16ac distribution patterns are not sufficient to specify H3K79 methylation zones

Because Dot1 is the sole H3K79 methyltransferase and no demethylase has been identified, we asked whether upstream regulatory factors could account for the zone-specific distributions of H3K79me3, H3K79me2, and H3K79me1. Trans-histone crosstalk between H2Bubi and H3K79 methylation, conserved from yeast to mammals, is required for efficient deposition of H3K79me2 and H3K79me3 [16–21]. H4K16ac has been reported to promote higher H3K79 methylation states by stabilizing the active state of Dot1 [22–24]. We therefore asked whether the distribution patterns of H2Bubi and H4K16ac explain the regional organization of the three H3K79 methylation states across the zones defined in Fig. 1.

**Figure 2.**
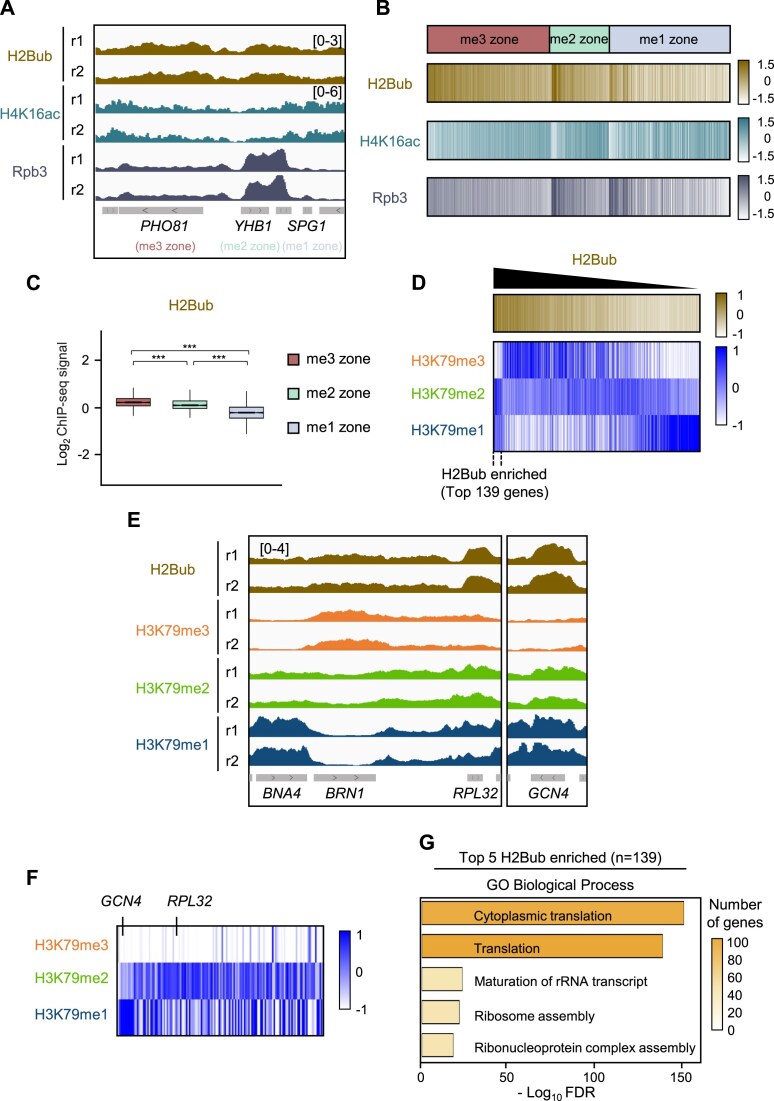
H2Bubi and H4K16ac distribution patterns do not correspond with the organization of the H3K79 methylation zones. (**A**) Genome browser tracks for H2Bubi (dark yellow, top) [54], H4K16ac (turquoise, middle) [22], and Rpb3 (dark gray, bottom) [37] from published datasets. The H2Bubi, H4K16ac, and Rpb3 signals were normalized to histone H2B, H3, and input, respectively. (**B**) Heatmaps showing the enrichment of H2Bubi, H4K16ac, and Rpb3 at the gene bodies in the me3, me2, and me1 zones. (**C**) Box plots of the log_2_ H2Bubi signals normalized to H2B in each zone. Statistical significance was calculated by the two-sided Wilcoxon rank-sum test (***P < 0.001). (**D**) Heatmaps of all genes containing three zones, when they were ordered according to descending H2Bubi signals at the gene body. H3K79 state signals were normalized to total H3. The data are averages of two biological replicates. There is a subset of 139 genes that show particularly high H2Bubi enrichment. (**E**) Representative loci illustrating the heterogeneous distributions of H2Bubi and the H3K79 methylation states: *BNA4* shows low H2Bubi and high H3K79me1, *BRN1* displays high H2Bubi and high H3K79me3, and *RPL32* and *GCN4* exhibit high H2Bubi, low H3K79me3, and high H3K79me2/ H3K79me1. (**F**) Heatmaps of the 139 genes that showed the strongest H2Bubi enrichment, as shown in (D). This subset largely lacked H3K79me3 but showed relative enrichment of H3K79me2 and/or H3K79me1. (**G**) GO Biological Process enrichment of the 139 genes in (D). The top five terms are shown; translation-related categories (e.g. GO:0002181, GO:0006412) are strongly enriched. Bar height represents the enrichment significance (−log10 *P*-value), and color intensity reflects the number of genes.

We first examined whether H2Bubi or H4K16ac correlated with any single H3K79 methylation zones. Published H2Bubi [54] and H4K16ac [22] ChIP-seq datasets were reprocessed using the same gene-level framework applied to our H3K79 methylation analyses in Fig. 1. The H2Bubi and H4K16ac distributions over the genes were assigned to each zone (Fig. 2A–C). Because H2Bubi is known to co-localize with H3K79me3 at many loci [15], we initially speculated that H2Bubi could be over-represented in the me3 zone. However, we found that representative genes from the me3 (*PHO81*), me2 (*YHB1*), and me1 (*SPG1*) zones displayed broadly similar H2Bubi levels (Fig. 2A, top). Comparisons of the H2Bubi heatmaps (Fig. 2B, top) and box plots of the H2Bubi read densities (Fig. 2C) in the zones also showed that H2Bubi was widely distributed across zones, although there was a modest H2Bubi elevation in the me3 zone.

To further investigate the relationship between H2Bubi and H3K79 methylation states, we ranked all genes by H2Bubi levels (high to low) and examined profiles of each H3K79 methylation state. As H2Bubi levels across gene bodies decreased, H3K79me3 levels declined, H3K79me2 levels remained relatively constant, and H3K79me1 levels increased (Fig. 2D). Interestingly, the genes most enriched in H2Bubi displayed very low H3K79me3 but relatively high H3K79me2 and H3K79me1 levels, suggesting that high H2Bubi does not uniformly promote H3K79me3 deposition. Rather, the heterogeneous relationship between H2Bubi and individual H3K79 methylation states likely underlies the zone-independent and broadly distributed pattern of H2Bubi observed across the genome, as shown in Fig. 2A–D. Individual loci further confirmed this heterogeneity across distinct zones. For example, the me1 zone gene *BNA4* had low H2Bubi and high H3K79me1, the me3 zone gene *BRN1* exhibited high H2Bubi together with high H3K79me3, and the me2 zone gene *RPL32* and the me1 zone gene *GCN4* both showed very high H2Bubi but low H3K79me3 (with high H3K79me2 and H3K79me1, respectively) (Fig. 2E).

We next focused on the 139 genes with the highest H2Bubi enrichment (Fig. 2F), which showed pronounced depletion of H3K79me3 and dominant enrichment of H3K79me2, with H3K79me1 appearing only at the very highest H2Bubi levels. GO analysis of these genes revealed significant enrichment for translation and ribosome assembly functions (Fig. 2G), suggesting that high H2Bubi at translation-related genes is associated with low H3K79me3 levels.

Similar to H2Bubi, H4K16ac also exhibited a broad distribution and no clear zone preference (Fig. 2A and B, middle; Supplementary Fig. S2A). Although H4K16ac has been reported to enhance Dot1 activity, its levels were generally anti-correlated with H3K79me3 levels (Supplementary Fig. S2B). Since both H2Bubi and H4K16ac are linked to transcription [20, 55, 56], we also examined the correspondence between the three zones and RNA Pol II (Rpb3) occupancy using our previous ChIP-seq dataset [37]. A subset of me2 zone genes showed high Rpb3 levels (Fig. 2A and B, bottom), but overall the zones did not differ substantially in median Rpb3 levels (Supplementary Fig. S2C). Interestingly, the 395 genes with the highest RNA Pol II occupancy resembled the top H2Bubi group: they lacked H3K79me3, bore higher H3K79me2 and/or H3K79me1 levels (Supplementary Fig. S2D, E), and were enriched for cytoplasmic translation categories (Supplementary Fig. S2F).

Taken together, these analyses indicate that while H2Bubi and H4K16ac are known to be important cofactors for Dot1-mediated methylation [16–22, 23], their genomic enrichment patterns and those of RNA Pol II cannot explain the discrete organization of the three H3K79 methylation zones. In addition, we identified a distinct subset of highly transcribed, translation-related genes that exhibit elevated H2Bubi and RNA Pol II levels but display low H3K79me3 and higher H3K79me2/H3K79me1 levels.

### Loss of H2Bubi disrupts the organization of the H3K79 methylation zones

**Figure 3.**
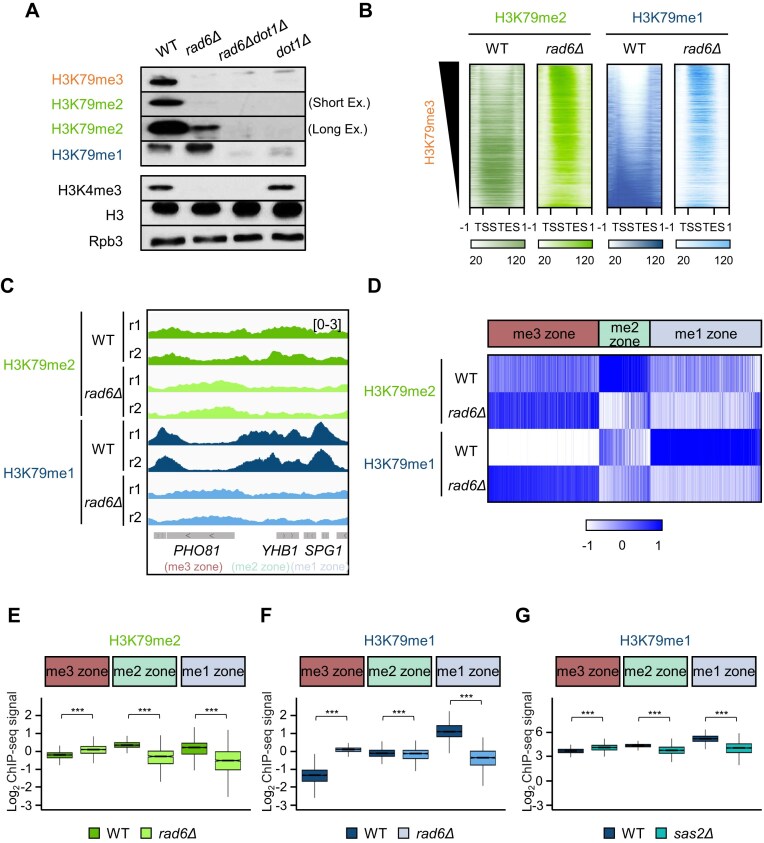
Rad6-mediated H2B ubiquitination is crucial for establishing H3K79 methylation zones. (**A**) Whole-cell extracts from strains were subjected to western blot analysis of the specified histone modifications. Histone H3 and Rpb3 served as loading controls. (**B**) Heatmaps of the H3K79me2 (WT, green; *rad6Δ*, light green) and H3K79me1 (WT, dark blue; *rad6Δ*, light blue) ChIP-seq signals across all 5750 genes, sorted by descending order of H3K79me3 signals in WT cells. The *y-axis* indicates each gene. The *x*-axis indicates the relative position from the TSS to the TES. The lower panels indicate the scale of RPM values from 20 to 120. (**C**) ChIP-seq tracks of H3K79me2 and H3K79me1 in the WT and *rad6Δ* strains. (**D**) Heatmaps showing the gene body enrichment of H3K79me2 (top) and H3K79me1 (bottom) at each zone in the WT and *rad6Δ* cells. (**E** and **F**) Box plots showing H3K79me2 (E) and H3K79me1 (F) levels at the gene bodies of each zone in the WT and *rad6Δ* strains. Values were normalized to histone H3 and displayed as log_2_ ratios. Significance levels were calculated by the Wilcoxon rank-sum test (***P < 0.001). (**G**) Box plots of the log_2_ H3K79me1 signals [22] normalized to H3 in WT and *sas2Δ* cells. ***P < 0.001 (Wilcoxon rank-sum test).

Although H2Bubi abundance did not associate strongly with the three H3K79 methylation zones (Fig. 2), this does not exclude the possibility that H2Bubi is important for establishing the H3K79 methylation zones. To test this, we eliminated H2Bubi by deleting *RAD6*, which encodes the E2 ubiquitin-conjugating enzyme required for ubiquitination of the K123 residue on H2B. Consistent with previous studies [16–20], western blot analysis showed a pronounced reduction of bulk H3K79me3 in *rad6Δ* mutants (Fig. 3A). Interestingly, while H3K79me2 was also reduced, a residual signal remained detectable upon longer exposure, and H3K79me1 levels were modestly increased. These observations led us to speculate that the global changes in H3K79 methylation may reflect a composite outcome of heterogeneous, gene-specific redistribution events of H3K79me1 and H3K79me2 rather than a uniform genome-wide decrease. To examine this possibility, we mapped the genome-wide distributions of H3K79me2 and H3K79me1 in WT and *rad6Δ* cells at all yeast genes (*n* = 5750) (Fig. 3B). Importantly, when *RAD6* was deleted, two unique trends emerged for H3K79me1: (i) a robust decrease at the genes where H3K79me1 normally predominates, and (ii) ectopic accumulation across gene bodies that are H3K79me3-enriched in WT cells (Fig. 3B). Similar but weaker patterns were observed for H3K79me2.

Consistent with these global trends, when we compared H3K79me2 and H3K79me1 signals between the WT and *rad6Δ* across each zone (Fig. 3C, D; Supplementary Fig. S3A), *rad6Δ* caused a marked loss of H3K79me2 and H3K79me1 at the me2 zone (e.g. *YHB1*) and the me1 zone (e.g. *SPG1*), respectively, while inducing ectopic H3K79me2 and H3K79me1 deposition at the me3 zone (e.g. *PHO81*). To quantify these zone-specific effects, the H3K79 state/H3 log_2_FC was determined for the H3K79me2 and H3K79me1 signals in each zone in the two strains. *rad6Δ* profoundly reduced H3K79me1 in the me1 zone (median log_2_FC = −1.44, ∼2.7-fold lower), slightly reduced H3K79me2 in the me2 zone (median log_2_FC = −0.601, ∼1.52-fold lower), strongly increased H3K79me1 in the me3 zone (median log_2_FC = +1.42, ∼2.7-fold higher), and modestly increased H3K79me2 in the me3 zone (median log_2_FC = +0.328, ∼1.26-fold higher) (Fig. 3E, F). Together, these results demonstrate that loss of H2Bubi does not cause a uniform depletion of H3K79 methylation but instead induces heterogeneous, state-specific redistribution of H3K79me1 and H3K79me2 across distinct chromatin zones.

Sas2-mediated H4K16 acetylation is thought to modulate H3K79 methylation [22, 23]. We thus asked whether this acetylation event also shapes the H3K79 methylation zones by deleting *SAS2. sas2Δ* slightly reduced H3K79me3 in the me3 zone while slightly increasing it in the me2 and me1 zones (Supplementary Fig. S3B, C). In addition, *sas2Δ* reduced H3K79me1 in the me1 zone (log_2_FC = −1.11, ∼2.17-fold lower) and modestly increased it in the me3 zone (log_2_FC = +0.415, ∼1.33-fold higher) (Fig. 3G; Supplementary Fig. S3D, E). Thus, *sas2Δ* induced similar H3K79 methylation pattern changes to *rad6Δ*, but the ectopic H3K79me1 gain in the me3 zone was substantially smaller in *sas2Δ* than in *rad6Δ* (Supplementary Fig. S3E).

Taken together, these results suggest that Rad6-mediated H2Bubi is the primary determinant of zone integrity: it establishes the zone-defining states and restricts aberrant H3K79me1 accumulation in the me3 zone. By contrast, Sas2-dependent H4K16ac fine-tunes H3K79 methylation patterns.

### Dot1 occupancy across H3K79 methylation zones is largely independent of H2Bubi

Dot1, unlike many other histone methyltransferases, methylates H3K79 via a non-processive (distributive) mechanism [57]. Thus, it seems possible that loss of H2Bubi profoundly altered the H3K79 methylation patterns in the three zones (Fig. 3) by causing the mislocalization of Dot1 on chromatin.

**Figure 4.**
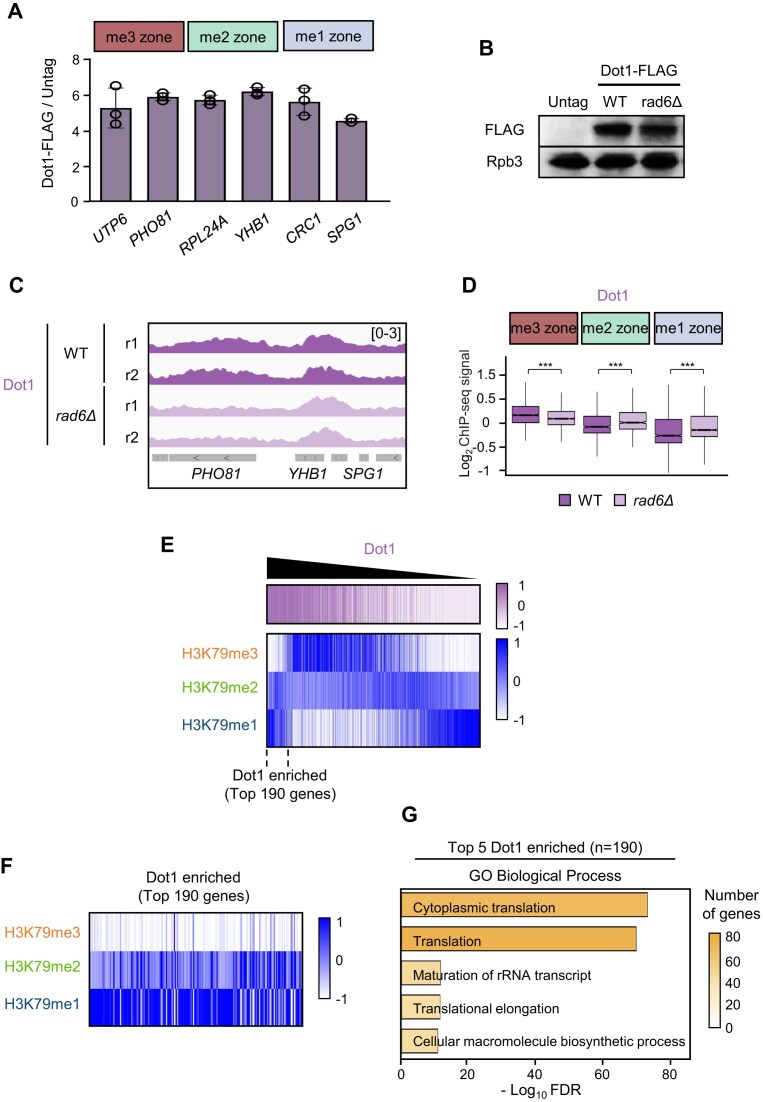
Dot1 occupancy patterns do not correspond to the state-specific H3K79 methylation zones. (**A**) ChIP with anti-FLAG antibody was performed with WT strains that did and did not express Dot1-FLAG. Analysis of typical me3, me2, and me1 zone genes showed that the Dot1-FLAG-bearing strain produced a clear ChIP signal relative to the untagged control. Dot1-FLAG enrichment was calculated as the ratio of Dot1-FLAG to untagged controls after normalization to input. Error bars show the standard deviation (SD) calculated from three biological replicates, each with three technical replicates. (**B**) Whole-cell extracts were subjected to western blot analysis with the indicated antibodies. Rpb3 was used as a loading control. (**C**) ChIP-seq tracks of Dot1 occupancy in the WT (purple) and *rad6Δ* (light purple) strains in two biological replicates (r1 and r2) normalized to input. (**D**) Box plots showing the Dot1 binding in the three zones in WT and *rad6Δ* cells, expressed as log_2_ levels of Dot1 occupancy normalized to input. ***P < 0.001 (two-sided Wilcoxon rank-sum test). The effect of *rad6Δ* on Dot1 binding patterns in the zones does not match the effect of *rad6Δ* on the methylation patterns in the zones shown in Fig. 3D–F. (**E**) Heatmaps showing the gene body enrichment of H3K79me3, H3K79me2, and H3K79me1 in genes sorted according to Dot1 signals in descending order. The data are averages of two biological replicates. The signals for H3K79 methylation states were normalized to the total H3 signal, and Dot1 signals were normalized to input. There is a subset of 190 genes that are highly enriched in Dot1. (**F**) Heatmaps showing the H3K79 methylation states in the 190 genes that had the highest Dot1 levels, as shown in (E). This outlier subset is largely depleted of H3K79me3 and shows relative enrichment of H3K79me2 and/or H3K79me1. (**G**) GO analysis for the 190 genes in (E). The top five terms are shown; translation-related categories are strongly enriched. Bar height represents the enrichment significance (−log_10_  *P*-value), and color intensity reflects the number of genes.

To test this, we generated WT and *rad6Δ* strains expressing FLAG-tagged Dot1 and performed ChIP using an anti-FLAG antibody. The Dot1-FLAG strain produces a clear signal relative to the untagged control (Fig. 4A). Unexpectedly, no strong zone-specific differences in Dot1 binding were detected by ChIP (Fig. 4A). After confirming that deletion of *RAD6* does not affect Dot1 protein abundance (Fig. 4B), we next carried out ChIP-seq analysis to compare Dot1 occupancy in WT and *rad6Δ* cells across the me3, me2, and me1 zones. At individual loci, Dot1 binding in the WT was relatively enriched in the me3 zone gene *PHO81* and the me2 zone gene *YHB1*, and *rad6Δ* decreased Dot1 occupancy in only the me3 zone gene *PHO81* (Fig. 4C). Genome-wide comparisons revealed that Dot1 binding was slightly higher at the me3 zone than in the me1 or me2 zone in WT cells. In *rad6Δ*, Dot1 occupancy was only modestly reduced in the me3 zone (Fig. 4D), a mild effect that does not match the striking loss of H3K79me3 in the me3 zone when *RAD6* was deleted (Fig. 3A). Moreover, Dot1 binding slightly increased in the me1 and me2 zones of *rad6Δ* cells (Fig. 4D), which is inconsistent with the significant reduction in H3K79me1 and H3K79me2 levels in their respective zones that was caused by *rad6Δ* (Fig. 3D–F). These results indicate that *rad6Δ* does not alter the H3K79 methylation patterns by inducing mislocalization of Dot1 on chromatin. Instead, H2Bubi likely regulates H3K79 methylation post-recruitment, possibly by modulating Dot1’s catalytic activity.

To further explore the disconnect between Dot1 occupancy and H3K79 methylation state, we analyzed individual methylation profiles. When genes were ranked by Dot1 occupancy from high to low, H3K79me3 levels generally decreased while H3K79me1 levels increased (Fig. 4E). Interestingly, a subset of 190 genes with the highest Dot1 occupancy deviated from this pattern: these genes were depleted of H3K79me3 but enriched for H3K79me2 and/or H3K79me1 (Fig. 4E, F). GO analysis revealed that this group is strongly enriched for functions related to cytoplasmic translation (Fig. 4G).

Together, these results demonstrate that Dot1 binding is largely independent of Rad6-mediated H2B ubiquitination and does not define H3K79 methylation zone identity. Furthermore, the enrichment of H3K79me2/H3K79me1, but not H3K79me3, at genes with high Dot1 occupancy suggests the existence of a distinct chromatin environment at translation-related genes that selectively restricts higher methylation states, likely through mechanisms beyond Dot1 recruitment.

### H3K79me3 and H3K79me1 have differential effects on gene expression dynamics

Previous studies have shown that loss of Dot1, the sole H3K79 methyltransferase in yeast, has little effect on transcription in steady-state conditions [22, 34]. We also observed this using a published dataset [34] (Supplementary Fig. S4A). Moreover, studies show that genome-wide levels of H3K79me3/me2/me1 correlate poorly with basal transcriptional output [13, 15, 20]. This, together with the fact that chromatin regulators often play important roles in regulating transcriptional responses to environmental changes [2, 34, 35, 36], suggests that H3K79 methylation marks may modulate transcriptional kinetics rather than steady-state expression.

**Figure 5.**
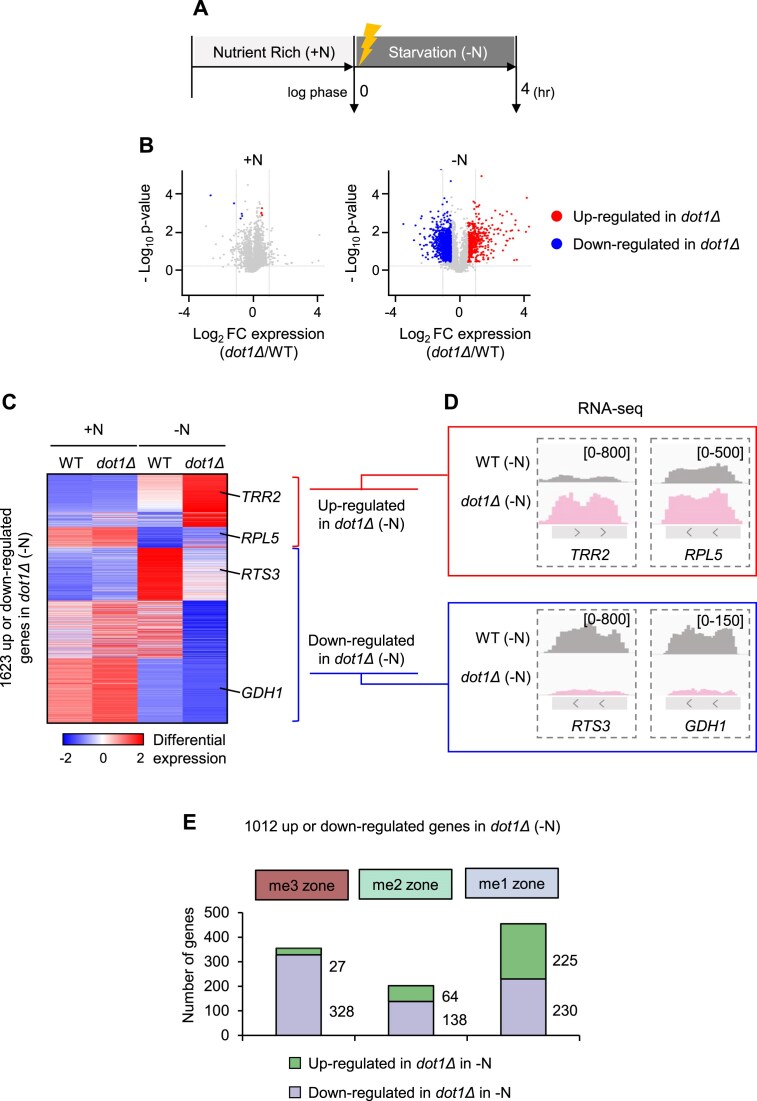
Loss of Dot1 globally alters gene expression during nutrient starvation. (**A**) Schematic depiction showing that WT and *dot1Δ* cells were first cultured in nutrient-rich (YPD; +N) medium and then transferred to nutrient-starvation conditions (0.15× YP; −N) for 4 h. The transcriptional responses were then assessed with RNA-seq. (**B**) Volcano plots of the RNA-seq data showing the differentially expressed genes in *dot1Δ* versus WT cells under +N (left) and −N (right) conditions. Red and blue indicate up-regulated and down-regulated genes in *dot1Δ*, respectively. Up-/down-regulation was defined as 1.4-fold change and FDR *q* < 0.5. (**C**) Heatmaps showing the transcript levels in WT and *dot1Δ* cells during +N (left) and −N (right) conditions for the 1623 genes that were differentially regulated by Dot1 in −N. (**D**) Genome browser view showing the mRNA levels of selected genes in the nutrient-starved WT (light gray) and *dot1Δ* (pink) cells. The selected genes are two that were up-regulated (*TRR2* and *RPL5*) and two that were down-regulated (*RTS3* and *GDH1*) in *dot1Δ* during −N. The *y*-axis indicates the mRNA read counts. (**E**) The number of the Dot1-regulated genes in −N (as determined by RNA-seq) that were located in the three H3K79 methylation zones. 92% of genes (328 out of 355) in the me3 zones were down-regulated in mutants for Dot1, and 71% of up-regulated genes (225 out of 316) in *dot1Δ* were enriched for H3K79me1.

To test this possibility, we compared transcriptional responses of WT and *dot1Δ* cells during nutrient starvation, a condition that induces global gene expression reprogramming [37]. Cells were grown in nutrient-rich medium (YPD; +N) and shifted to nutrient-depleted medium (0.15× YP; −N) for 4 h (Fig. 5A). Under these conditions, WT cells exhibited extensive transcriptional changes, with 2261 genes significantly induced and 1463 genes repressed (Supplementary Table S4). RNA-seq analyses showed that *DOT1* deletion had minimal effects on basal expression levels under nutrient-rich conditions (Fig. 5B, left) but greatly altered expression in nutrient-starved conditions (Fig. 5B, right). This suggests that Dot1-mediated H3K79 methylation may be critical for orchestrating transcriptional responses to environmental changes.

We identified the 1623 genes that were up-regulated (*n* = 478, e.g. *TRR2* and *RPL5*) or down-regulated (*n* = 1145, e.g. *RTS3* and *GDH1*) in *dot1Δ* cells by nutrient starvation (Fig. 5C, D; Supplementary Table S5) and asked whether these genes were located preferentially in any of the H3K79 methylation zones. For this, we integrated the RNA-seq results with our ChIP-seq-based zone assignments. We found that of the 1623 Dot1-regulated genes in −N, 1012 were located in one of the three zones: specifically, 355, 202, and 455 were in me3, me2, and me1, respectively (Fig. 5E). Strikingly, of the 355 genes in the me3 zone, 328 (92%) were down-regulated (Fig. 5E; Supplementary Fig. S4B), suggesting a positive role for H3K79me3 in transcription. Moreover, of the 202 Dot1-regulated genes in the me2 zone, 138 (68%) were down-regulated (Fig. 5E; Supplementary Fig. S4B). Notably, of 316 genes up-regulated in *dot1Δ*, 225 genes (71%) were enriched for H3K79me1, suggesting a negative effect of H3K79me1 in transcription, even though ~50% (230/455) of genes in the me1 zone were also down-regulated in mutants for Dot1 (Fig. 5E; Supplementary Fig. S4B). This suggests that H3K79me1 can exert dual, context-dependent effects: repressive for some loci yet supportive for others. Comparison with starvation-responsive genes in WT cells suggested a zone-specific pattern. Genes down-regulated in *dot1Δ* cells were enriched in the me3 zone and largely overlapped with genes repressed upon nutrient starvation. In contrast, me1-zone genes were distributed across repressed, stable, and induced categories, with a bias toward starvation-induced genes (Supplementary Fig. S4C).

Together, these findings suggest that H3K79me3 and H3K79me1 may have distinct roles during nutrient starvation-induced transcriptional remodeling.

### H3K79 methylation zones are broadly maintained during transcriptional reprogramming

**Figure 6.**
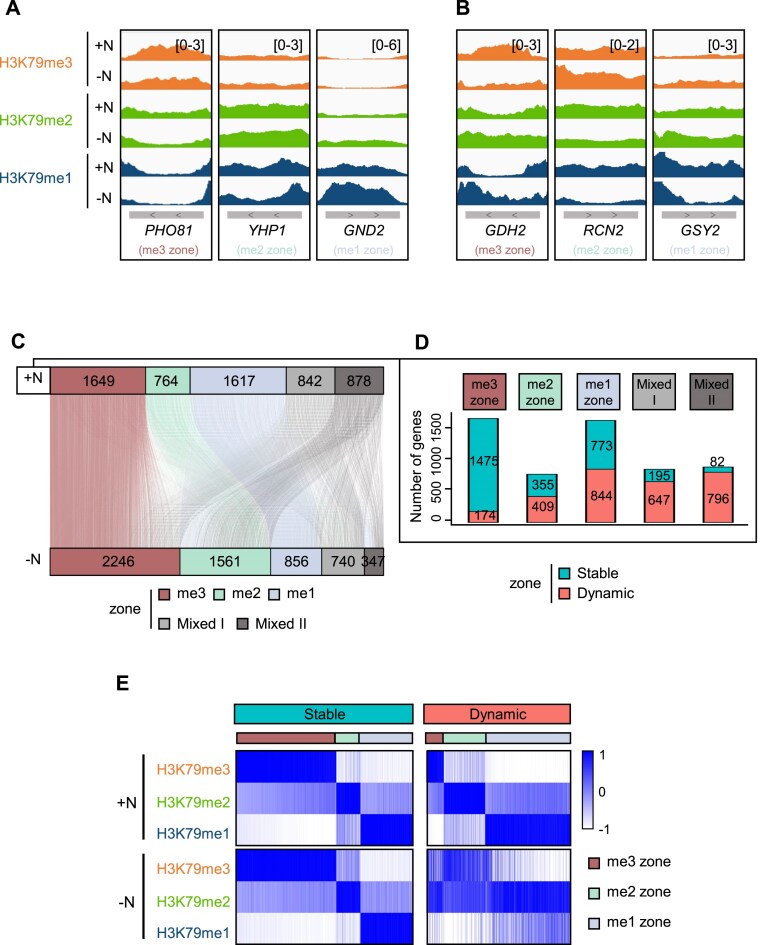
Effect of nutrient starvation on H3K79 methylation zone stability. (**A** and **B**) Genome browser tracks of representative genes that showed nutrient stress-induced changes in H3K79 methylation. The genes either (A) maintained their predominant state (*PHO81, YHP1*, and *GND2*) or (B) changed their predominant state (*GDH2, RCN2*, and *GSY2*). The ChIP-seq signals for H3K79me3 (orange), H3K79me2 (green), and H3K79me1 (dark blue) in +N and −N conditions are shown. (**C**) Sankey plot illustrating the changes between the zones in the +N and −N conditions. Each line represents a gene and is colored according to its zone classification (me3, me2, me1, Mixed I, Mixed II). (**D**) The number of genes in the +N H3K79 methylation zones that underwent stable or dynamic changes in −N conditions. The genes were classified as stable if they retained their +N zone classification under −N, and dynamic if they switched to another zone. (**E**) Heatmaps showing the H3K79me3, H3K79me2, and H3K79me1 enrichment across gene bodies in +N and −N conditions. While the H3K79 methylation zone genes generally remained stable under nutrient stress, a subset underwent state conversion.

Although H3K79 methylation has generally been considered a relatively stable modification [36], we discovered that Dot1-mediated H3K79 methylation plays an active role in dynamic transcriptional responses (Fig. 5). This prompted us to ask whether the state-specific H3K79 methylation zones defined under steady-state conditions are preserved or reorganized during transcriptional reprogramming. To address this, we profiled genome-wide distributions of H3K79me3, H3K79me2, and H3K79me1 in cells grown in nutrient-rich medium (+N) and after 4 h of nutrient starvation (−N) (Fig. 5A). Analysis of individual genes showed that nutrient starvation induced two common types of responses, both observed across the three zones. Specifically, genes either remained within their steady-state H3K79 methylation zone despite changing their signal intensity (e.g. *PHO81, YHP1*, and *GND2*) (Fig. 6A) or switched their predominant state. For example, the me3-zone gene *GDH2* lost H3K79me3 while gaining lower methylation states, the me2-zone gene *RCN2* lost H3K79me2 but gained H3K79me3, and the me1-zone gene *GSY2* lost H3K79me1 while acquiring higher methylation states (Fig. 6B).

To systematically assess how the H3K79 methylation zones changed during nutrient starvation, we reclassified all genes in −N conditions into three zones using the same criteria applied under +N. Comparison of the +N and −N classifications (Fig. 6C) showed that the majority of the genes (65%) retained their original zone (stable group) (Fig. 6D, E; Supplementary Fig. S5A; Supplementary Table S6) and the remaining 35% changed zone (dynamic group) (Fig. 6D, E; Supplementary Fig. S5A; Supplementary Table S6). The me3-zone genes were particularly likely to be stable, while the me2-zone and me1-zone genes were equally likely to be stable and dynamic. Interestingly, the zone conversions exhibited a directional bias toward the me2 zone: genes originating in either the me1 or me3 zone preferentially shifted into H3K79me2-enriched regions under −N (Fig. 6E; Supplementary Fig. S5B). We next asked whether these conversions were driven by transcriptional reprogramming during nutrient starvation by examining their RNA Pol II occupancy using our previous ChIP-seq dataset [37]. In the me3 zone, both stable and dynamic groups showed reduced RNA Pol II occupancy, with a more pronounced decrease in the dynamic group. In the me1 zone, both groups exhibited similarly increased RNA Pol II occupancy under −N (Supplementary Fig. S5C), suggesting that transcriptional activity generally did not account for the zone reorganization.

These findings indicate that H3K79 methylation zones are globally stable during environmental changes, but a subset of genes is reorganized into other zones, with a preference towards enrichment of the H3K79me2 state.

## Discussion

**Figure 7.**
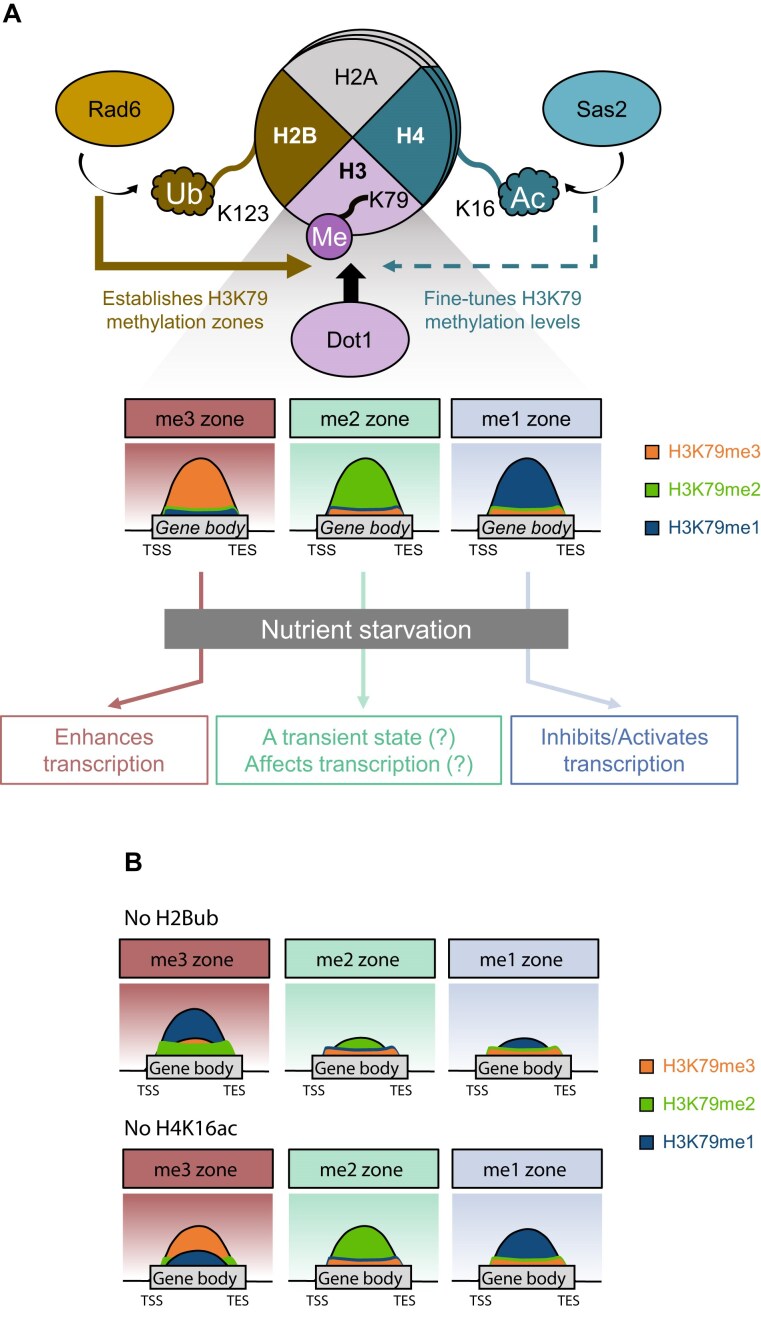
Model for the establishment of the three state-specific H3K79 methylation zones and their role in transcription. (**A**) H3K79me3, H3K79me2, and H3K79me1 predominantly occupy distinct subsets of genes, forming three distinct chromatin zones that largely remain stable, even under nutrient starvation. The zones are generated by crosstalk between Rad6-mediated H2Bubi and Sas2-mediated H4K16ac that regulates Dot1 activity. The zones associate with distinct transcriptional effects: H3K79me3 supports transcriptional activation, whereas H3K79me1 may contribute to gene repression or activation depending on context under nutrient starvation conditions. A subset of genes shifts from the me1 or me3 zones into the me2 zone, suggesting that the me2 zone may represent a transient chromatin state during epigenomic reorganization. Alternatively, H3K79me2 itself may contribute directly to transcriptional regulation. (**B**) Rad6-mediated H2Bubi is essential for establishing the organization of genes in the three H3K79 methylation zones: its absence markedly alters the H3K79 methylation patterns. In particular, H2Bubi prevents the aberrant accumulation of H3K79me1 in the me3 zone. By contrast, loss of Sas2-mediated H4K16ac has much less profound effects on the global distribution of H3K79 methylation, indicating that H4K16ac acts primarily as a fine-tuner of H3K79 methylation levels.

Histone H3 lysine 79 (H3K79) can exist in three methylation states (me1, me2, and me3), yet how these modifications are distributed across the genome and functionally interpreted has remained unresolved. Previous studies reported that all three states exhibit similar distributions along gene bodies, supporting a model of functional redundancy [20, 57]. However, our study reveals that H3K79me1, H3K79me2, and H3K79me3 define state-specific “methylation zones” that contain distinct gene subsets (Fig. 7). We also showed that this pattern is established by trans-histone crosstalk. Specifically, Rad6-mediated H2B ubiquitination is the primary determinant of zone establishment, and Sas2-mediated H4K16 acetylation fine-tunes the methylation levels within the zones. Functionally, these zones associate H3K79 methylation states with distinct transcriptional roles: H3K79me3 is linked to gene activation, while H3K79me1 associates with either gene repression or activation, depending on the context.

The importance of Rad6-mediated H2B ubiquitination in the establishment of H3K79 methylation zones is consistent with many studies showing that this trans-histone crosstalk is essential for the proper deposition of H3K79me2 and H3K79me3 in yeast and mammals [16–21]. It has been widely proposed that greater H2Bubi abundance promotes the formation of H3K79me3 and distinguishes it from H3K79me2 [15]. However, this notion has been questioned by several studies demonstrating that H2Bubi levels do not necessarily correlate with H3K79me3 abundance [58, 59]. For example, *CHD1* deletion drastically reduces H2Bubi, yet the genome-wide H3K79me3 patterns remain largely intact [60], indicating that even minimal H2Bubi levels can generate a largely normal H3K79me3 distribution *in vivo*. Moreover, *UBP8* deletion, which increases H2Bubi, does not elevate global H3K79me3 [61], and *H2B-T122R/D* mutants that either decrease or increase H2Bubi have little effect on H3K79me2/3 [62]. Consistent with these findings, our analyses show that H2Bubi abundance *per se* does not predict the distribution of H3K79 methylation zones (Fig. 2). Instead, the presence or absence of H2Bubi, rather than its quantitative abundance, is likely critical for establishing zone identity (Fig. 7B).

In addition to establishing normal H3K79me2 and H3K79me3 patterns, Rad6-dependent H2B ubiquitination is also a key regulator of H3K79me1 distribution [16–20]. While loss of Rad6 did not markedly change global H3K79me1 levels [20] (Fig. 3A), we did observe that loss of Rad6 profoundly reduced H3K79me1 in the me1-zone genes. Importantly, loss of Rad6 also induced ectopic gain of H3K79me1 in the me3 zone. Thus, globally unchanged H3K79me1 levels in mutants for Rad6 likely reflect opposing changes: a decrease of H3K79me1 in the me1 zone is balanced by the gain of H3K79me1 in the me3 zone. This leads to an important question: where does the increased H3K79me1 in the me3 zone originate? A clue to the answer may be that *RAD6* deletion not only concomitantly (i) depletes H3K79me1 at the gene bodies in the me1 zone and (ii) increases H3K79me1 at the gene bodies of the me3 zone, but also (iii) decreases H3K79me1 in the flanking regions of the me3 zone (Fig. 1F; Supplementary Fig. S3A). Thus, the increased H3K79me1 in the me3 zone upon *RAD6* deletion could come from redistribution/expansion of the flanking H3K79me1 marks onto the gene bodies. However, the possibility of *de novo* H3K79me1 gain at the gene bodies in the me3 zone cannot be excluded either. If H3K79me1 is redistributed, further studies are needed to determine how Rad6-mediated H2Bubi constrains this and to elucidate the biological significance of the flanking H3K79me1 marks in the me3 zone.

Our data further indicate that H2Bubi does not establish zone specificity by recruiting Dot1, because loss of H2Bubi does not significantly alter Dot1 occupancy (Fig. 4D). In fact, upon loss of Rad6, Dot1 binding increased slightly in the me1 and me2 zones despite the marked loss of H3K79me1 and H3K79me2 in those zones. This indicates that H2Bubi regulates genome-wide H3K79 methylation patterns independently of Dot1 recruitment, most likely by modulating its catalytic activity after recruitment. This interpretation is supported by cryo-electron microscopy studies showing that H2Bubi and H4K16ac cooperate to allosterically regulate Dot1 activity: specifically, H2Bubi restricts Dot1 conformation on the nucleosome, and H4K16ac constrains and stabilizes its active state [23, 24]. Consistent with this, our data show that deletion of *SAS2*, which encodes the H4K16 acetyltransferase, altered the relative levels of the three H3K79 methylation states in a manner similar to *rad6Δ*, but with far smaller magnitude (Fig. 3G; Supplementary Fig. S3C–E). These findings suggest that H2Bubi acts as the primary determinant of H3K79 methylation patterns, whereas Sas2-dependent H4K16ac fine-tunes methylation levels by stabilizing Dot1’s active conformation.

Whether H3K79 methylation shapes transcription, or vice versa, has been a longstanding question. Previous studies reported weak genome-wide correlations between H3K79 methylation and transcriptional activity [2, 34–37]. Similarly, we could not find any strong evidence suggesting that transcriptional activity determines the distinct H3K79 methylation zones (Fig. 2B; Supplementary Fig. S2C, D). Rather, our data suggest that the H3K79 methylation states themselves differentially regulate transcription, particularly during environmental transition. Specifically, although we (Fig. 5B; Supplementary Fig. S4A) and others [22, 34] found that loss of Dot1 had little effect on steady-state expression, we observed that (i) Dot1 greatly alters transcription during nutrient starvation (Fig. 5B, C) and (ii) the transcriptional effects differ depending on the H3K79 methylation state: H3K79me3 is consistently associated with transcriptional activation, whereas H3K79me1 can promote either activation or repression with roughly equal frequency (Figs 5E and 7). The determinants that underlie dual transcriptional roles of H3K79me1 remain to be elucidated. Moreover, future studies employing locus-specific epigenome editing will be important to identify the precise mechanisms by which the H3K79 methylation states differentially regulate transcription [63]. Although reader proteins that directly recognize H3K79 methylation have not yet been identified, and it is not clear whether they actually exist [32, 33], studies searching for reader proteins are warranted because they are likely to function in a context- and locus-specific manner. For example, they may selectively recognize particular H3K79 methylation states only in the presence of additional chromatin features or during specific transcriptional transitions [51, 52].

Another insight from our study is that two-thirds of the genes in the three H3K79 methylation zones retained their zone identity during transcriptional reprogramming induced by nutrient starvation, despite changes in methylation intensity (Fig. 6). In contrast, the remaining one-third of the genes in the zones switched their zone identity, with the most common changes being the conversion of me1 and me3 zones into the me2 zone (Fig. 6E; Supplementary Fig. S5B). This enrichment of transitions toward the me2 zone suggests that it may represent a transient chromatin state during dynamic epigenomic reorganization, although whether H3K79me2 itself plays an active role in transcriptional regulation remains unclear (Fig. 7). The conversion of H3K79me3 into H3K79me2 further implies that such reorganization could be shaped by context-dependent H3K79 demethylases, although putative demethylases remain to be identified. Notably, comparison of the stable and dynamic gene groups revealed no obvious differences in RNA Pol II occupancy (Supplementary Fig. S5C), suggesting that RNA Pol II transcription or transcription-coupled histone turnover is unlikely to drive the conversion from H3K79me3 to H3K79me2. Furthermore, although our findings support a key role for H2B ubiquitination in maintaining zone integrity under steady-state conditions, it remains to be determined whether H2B ubiquitination is also broadly required for the establishment or reconfiguration of these zones upon transcriptional reprogramming.

Interestingly, a distinct subset of genes associated with translation and high expression levels displayed an atypical pattern of H3K79 methylation (Figs 2 and 4; Supplementary Fig. S2). Despite exhibiting the highest levels of Dot1 binding, H2Bubi, and RNA Pol II occupancy, these genes were markedly depleted of H3K79me3. Instead, they were enriched for H3K79me2 and/or H3K79me1. These observations raise an important question: why is H3K79me3 selectively absent from these genes despite robust Dot1 recruitment? One possibility is that these loci have a distinct chromatin environment that actively inhibits the conversion to H3K79me3 by Dot1, potentially through local histone composition, nucleosome dynamics, or chromatin-associated factors. Alternatively, these genes may be subject to active demethylation by as yet unidentified H3K79me3 demethylases. It is also possible that Dot1 may have regulatory functions at these loci independent of its catalytic activity [22, 54], potentially through interactions with other chromatin-modifying complexes or transcriptional machinery.

Taken together, our findings reveal the genome-wide organization of genes into three zones that are characterized by high levels of each H3K79 methylation state. We also show that this organization may be driven by trans-histone crosstalk involving H2B ubiquitination, which serves as the primary determinant, and H4K16 acetylation, which acts to fine-tune methylation. Given that Dot1-mediated H3K79 methylation is highly conserved from yeast to humans, these findings raise the possibility that similar state-specific chromatin partitioning mechanisms may exist in mammalian genomes. This is particularly relevant in the context of human disease, as the mammalian homolog DOT1L has been implicated in human diseases, including MLL-rearranged leukemia [24, 25, 64]. If H3K79 methylation states are differentially distributed across gene subsets in the human genome, the functional alterations of DOT1L in leukemia may lead to leukemogenesis by reconfiguring the H3K79 methylation zones and their associated transcriptional outputs.

## Supplementary Material

gkag291_Supplemental_Files

## Data Availability

Gene Expression Omnibus (GEO) accession numbers: GSE310166 and GSE310163.
