# Supplementary material for: Trans-histone crosstalk establishes distinct H3K79 methylation zones with differential transcriptional functions
Source: Nucleic Acids Res. 2026 Apr 2;54(6):gkag291. doi: 10.1093/nar/gkag291 (PMC13044951; doi:10.1093/nar/gkag291)
Supplement: gkag291_Supplemental_Files [file gkag291_supplemental_files.zip › Supplementary Figures with legends, Table S1-2.pdf]

Supplementary Figure S1

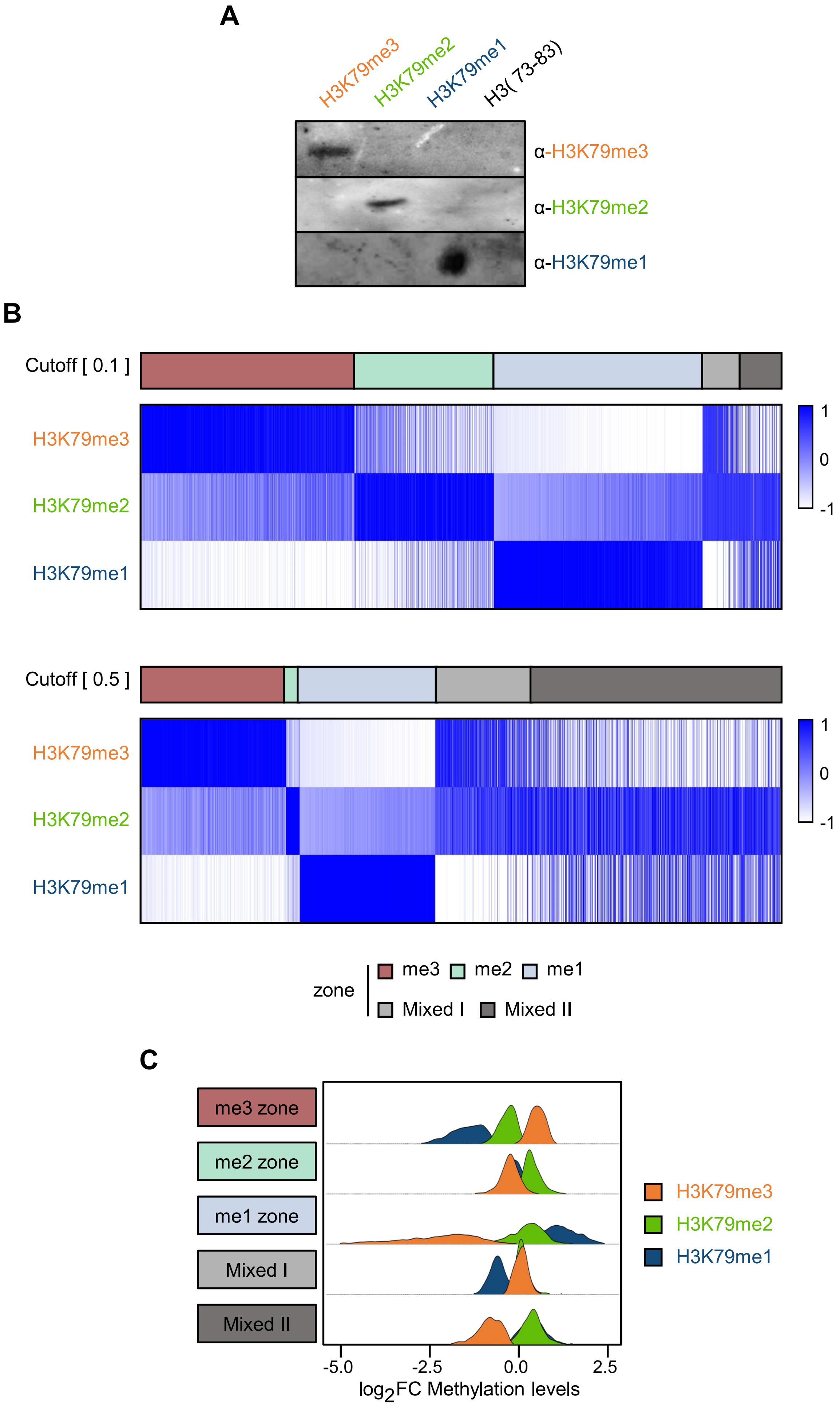

Supplementary Figure S2

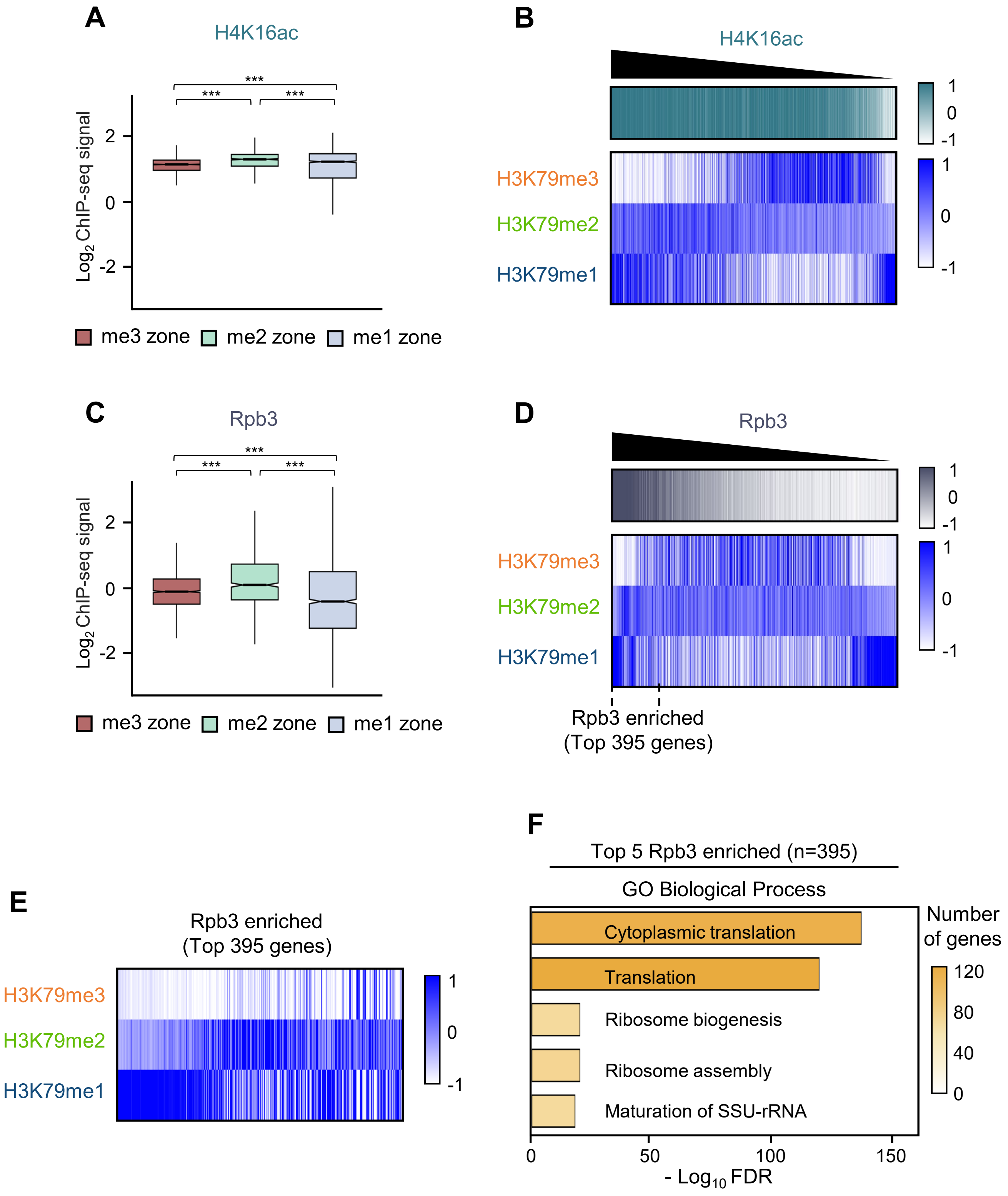

Supplementary Figure S3

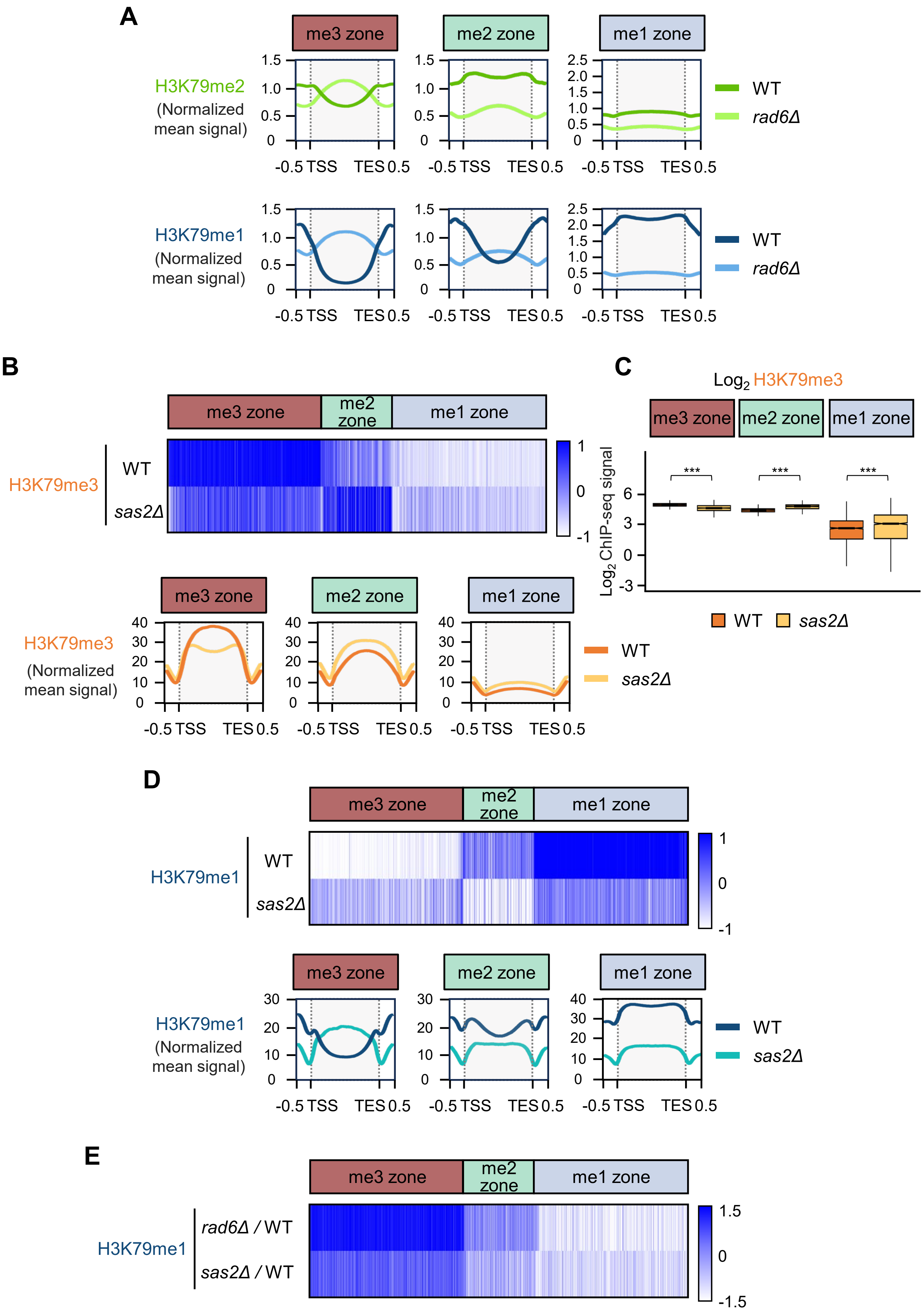

Supplementary Figure S4

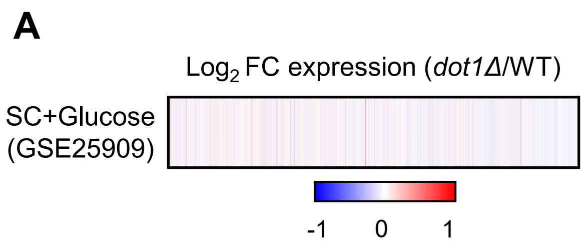

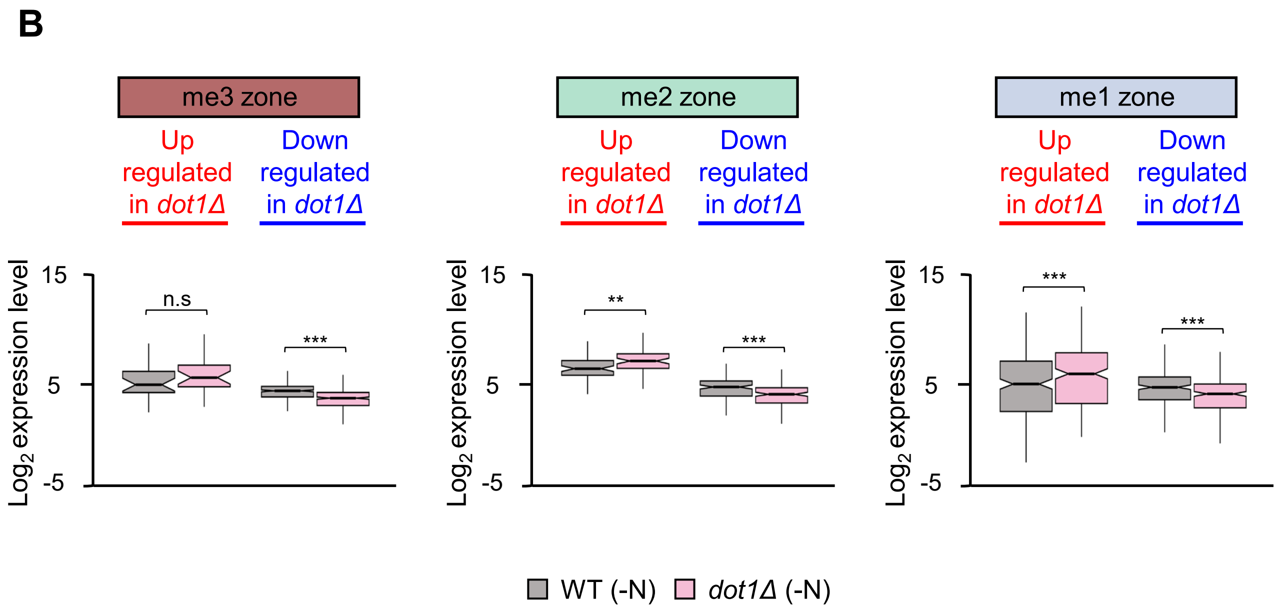

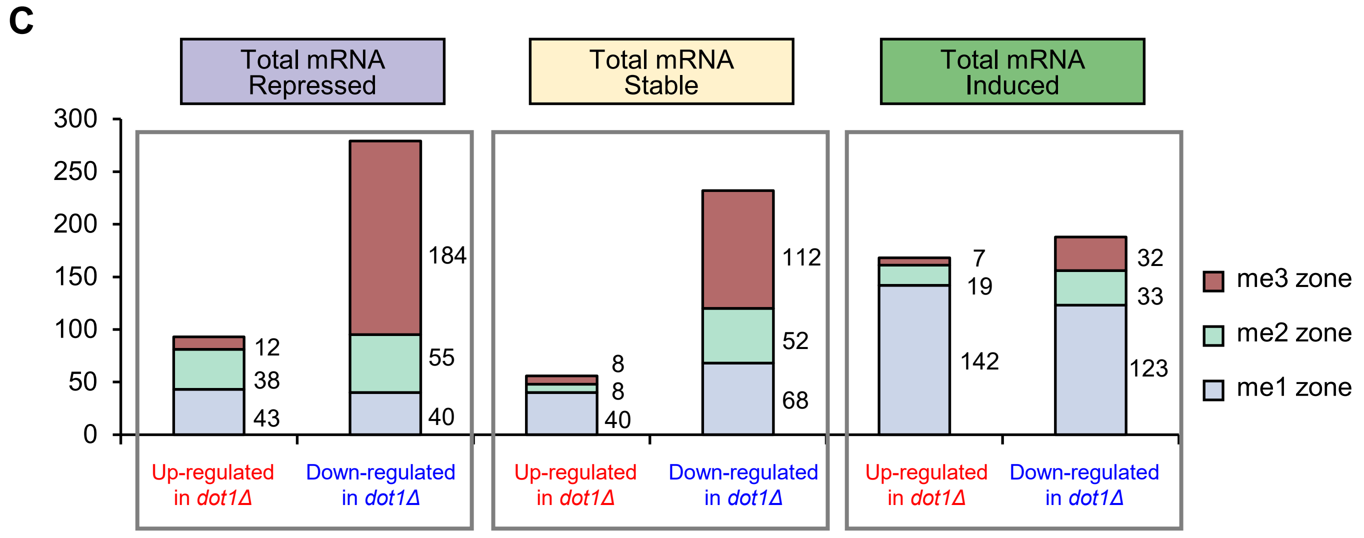

Supplementary Figure S5

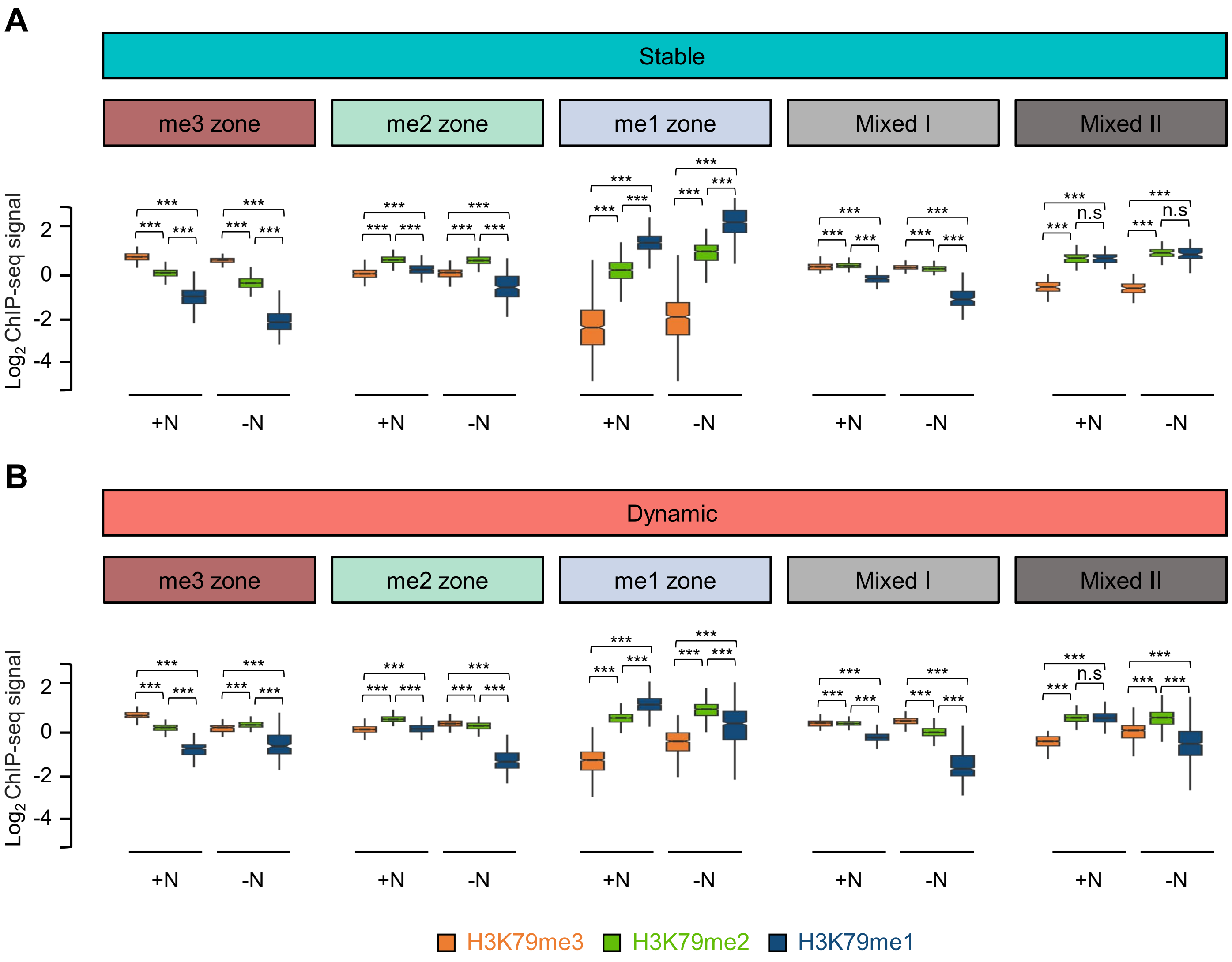

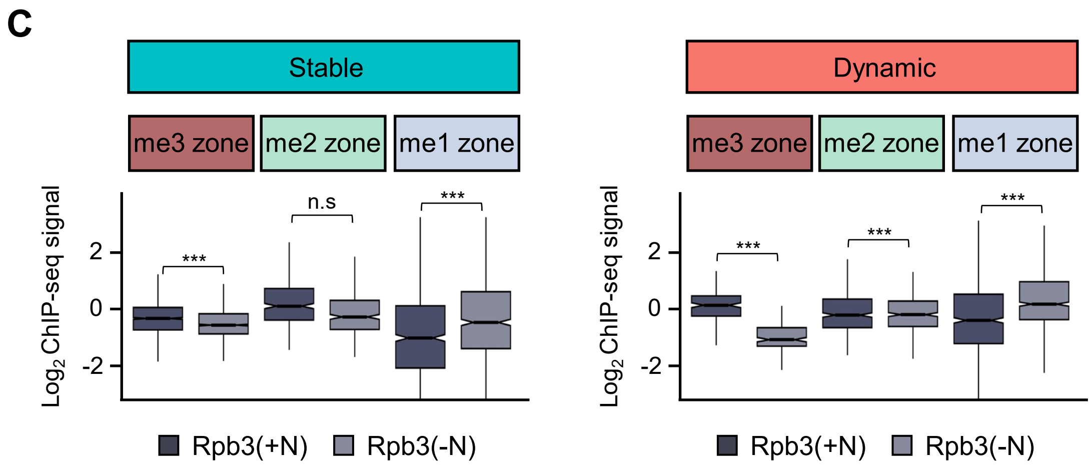

Supplementary Figure S1. State-specific distribution profiles of H3K79 methylation.

(A) Antibody specificity validation. Histone peptides, H3, mono-, di-, or tri-methylated H3K79, were subjected to western blot analyses with the indicated antibodies used in this study. **(B)** Heatmaps of gene body enrichment of H3K79 methylation states for genes assigned to each zone. Alternative log_2 fold-change thresholds (0.1 or 0.5) altered only the number of genes assigned to each zone but did not affect the overall zonal pattern. **(C)** Ridge plots illustrate the quantitative relationships among H3K79me3, H3K79me2, and H3K79me1 across the defined H3K79 methylation zones.

Supplementary Figure S2. H4K16ac and RNA Pol II occupancy are not sufficient to specify H3K79 methylation zones.

(A) Box plots of the log_2 H4K16ac signal (Lee et al., 2019) normalized to H3 within each zone. Statistical significance was calculated by a two-sided Wilcoxon rank-sum test (***P < 0.001). **(B)** Genes ordered by decreasing H4K16ac. Heatmaps show gene-body enrichment of H3K79me3, H3K79me2, and H3K79me1. Signals for H3K79 states and H4K16ac were normalized to the total H3 signal. A modest inverse trend between H4K16ac and H3K79me3 is apparent. **(C)** Box plots of the log_2 Rpb3 signal (Lee et al., 2025) normalized to input within each zone. Statistical significance was calculated by the two-sided Wilcoxon rank-sum test (***P < 0.001). **(D)** Genes ordered by decreasing RNA Pol II (Rpb3) occupancy. Heatmaps as in (B) for H3K79me3, H3K79me2, and H3K79me1. **(E)** Heatmaps for the top 395 Rpb3-enriched genes identified in (D). This outlier subset is largely depleted of H3K79me3 and shows relative enrichment of H3K79me1 and/or H3K79me2. **(F)** Gene Ontology (GO) analysis for the 395 genes in (D). The top five terms are shown; translation-related categories are strongly enriched. Bar height represents the enrichment significance (−log10 p-value), and color intensity reflects the number of genes.

Supplementary Figure S3. Effects of Rad6-dependent H2B ubiquitination and Sas2-mediated H4K16 acetylation on H3K79 methylation zone integrity

(A) Average plots of H3K79me2 (WT, green; *rad6Δ*, light green) and H3K79me1 (WT, dark blue; *rad6Δ*, light blue) across genes in each zone. Gene bodies are shaded. **(B)** Heatmaps and average profiles (gene bodies are shaded) of H3K79me3 (Lee et al., 2019) in WT (orange) and *sas2Δ* (yellow) across gene bodies at each zone. **(C)** Box plots of log_2 H3K79me3 signal normalized to H3 in WT and *sas2Δ* at each zone. ***P < 0.001 (Wilcoxon rank-sum test). **(D)** Heatmaps and average profiles of H3K79me1 (Lee et al., 2019) in WT (dark blue) and *sas2Δ* (cyan) across gene bodies at each zone. Gene bodies are shaded. **(E)** Heatmaps of H3K79me1 log_2-fold changes in *rad6Δ* (top) and *sas2Δ* (bottom) mutants relative to WT.

Supplementary Figure S4. Dot1-dependent transcriptional responses are likely zone-dependent under nutrient starvation.

(A) Heatmap of microarray data from Lenstra et al., 2011 showing minimal changes in gene expression upon *DOT1* deletion under steady-state conditions. SC indicates synthetic complete medium. **(B)** Box

plots of $\log_2$ expression levels under -N condition for *dot1Δ*-regulated genes (as defined in Fig. 5C), grouped by H3K79 methylation zones, shown for WT and *dot1Δ*. Statistical significance was assessed with the two-sided Wilcoxon rank-sum test (***P* < 0.01, ****P* < 0.001, n.s: not significant). **(C)** Distribution of Dot1-regulated genes across transcriptional response categories (repressed, stable, and induced) within each H3K79 methylation zone under nutrient starvation conditions. Genes were classified as repressed or induced based on a ≥1.6-fold change with FDR *q* < 0.5.

Supplementary Figure S5. H3K79 methylation zones are broadly stable, with a dynamic subset upon nutrient starvation.

(A, B) Box plots of per-gene $\log_2$-transformed normalized ChIP-seq signals for H3K79me3, H3K79me2, and H3K79me1 in +N and −N, shown separately for genes classified as (A) stable (zone preserved in −N) and (B) dynamic (zone changed in −N), using the same zone-assignment criteria as in +N. Statistical significance between conditions was assessed with the two-sided Wilcoxon rank-sum test (****P* < 0.001, n.s: not significant). **(C)** Rpb3 occupancy changes under −N do not differ significantly between the Stable and Dynamic groups within each zone. (****P* < 0.001, n.s: not significant).

Supplementary Table S1. Strains used in this study.

Strain	Genotype	Source or Reference
BY4741	MATa, ura3Δ0, leu2Δ0, his3Δ1, met15Δ0	SGD www.yeastgenome.org
YF534	MATa, ura3Δ0, leu2Δ0, his3Δ1, met15Δ0, rad6Δ::KanMX4	Saccharomyces Genome Deletion Project
YTK1644	MATa, ura3Δ0, leu2Δ0, his3Δ1, met15Δ0, Dot1-3xFLAG::KanMX6	This study
YTK1677	MATa, ura3Δ0, leu2Δ0, his3Δ1, met15Δ0, Dot1-3xFLAG::KanMX6, rad6Δ::URA3	This study
YTK251	MATa, ura3Δ0, leu2Δ0, his3Δ1, met15Δ0, dot1Δ::KanMX4	This study

Supplementary Table S2. Oligonucleotides used in this study.

Oligo Name	Sequences (5' - 3')	Purpose
Dot1_3xFLAG	(F) GCAAGAGGTAGGAGGAACAGAGGTACGCCGGTG AAGTATACCAGCGGATCCCCGGGTTAATTAA (R) CTACTTAGTTATTCATACTCATCGTTAAAAGCCGTT CAAAGTGCCGAATTCGAGCTCGTTTAAAC	Dot1-Ct-3xFLAG
Dot1_KANMX4_F	(F) CACCAGTAATTGTGCGCTTTGGTTACATTTTGTT GTACAGTA AGATTGTACTGAGAGTGCAC	Knockout
Dot1_KANMX4_R	(R) CTACTTAGTTATTCATACTCATCGTTAAAAGCCGTT CAAAGTGCC CTGTGCGGTATTTCACACCG	Knockout
Rad6_URA3_F	(F) GAATTCCAAAGATTATTTTTAGGCAGACAGAGACT AAAAGATAAAGCGTC TTCAATTCAATTCATCATTT	Knockout
Rad6_URA3_R	(R) AATTCATAATATCGGCTCGGCATTCATCATTAAGAT TCTTTTGATTTTTC CGCAGGGTAATAACTGATAT	Knockout
UTP6	(F) CGAGCTGAATTTCATCACTAAGT (R) CCAGTGGATGGAACTTGTAAA	ChIP-qPCR
PHO81	(F) CACGCTTATTTATTCTAGATGAGGC (R) TCCAGAATTGGCTTTTCAATGGG	ChIP-qPCR
RPL24A	(F) AAATCGATTCTTTTTCAGGT (R) CTGAATAAGACAGTCCAAGC	ChIP-qPCR
YHB1	(F) GCAATTGCTGACATCTTCATCAC (R) GCGTCCTTGTGTAAATATTCAGAGA	ChIP-qPCR
CRC1	(F) CATTCAGGCGGCTAAAACCATTG (R) CGTTCAAGATATTGACCGGTTCATC	ChIP-qPCR
SPG1	(F) CGCTGCTGTGGTACCGACCGC (R) CATCATCTCTTGTAAACTCTTACC	ChIP-qPCR
